# Correction: The VertiGO! Trial protocol: A prospective, single-center, patient-blinded study to evaluate efficacy and safety of prolonged daily stimulation with a multichannel vestibulocochlear implant prototype in bilateral vestibulopathy patients

**DOI:** 10.1371/journal.pone.0317175

**Published:** 2025-01-03

**Authors:** Bernd L. Vermorken, Benjamin Volpe, Stan C. J. van Boxel, Joost J. A. Stultiens, Marc van Hoof, Rik Marcellis, Elke Loos, Alexander van Soest, Chris McCrum, Kenneth Meijer, Nils Guinand, Angélica Pérez Fornos, Vincent van Rompaey, Elke Devocht, Raymond van de Berg

The images for Figs 2 and 4 are incorrectly switched. The image that appears as Fig 2 should be Fig 4, and the image that appears as Fig 4 should be Fig 2. The figure captions appear in the correct order.

**Fig 2:**
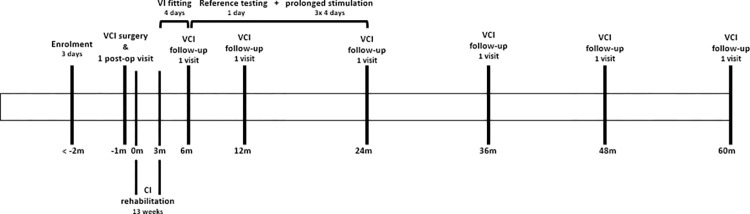
Timeline per subject of the VertiGO! trial. Timeline in months (m). VCI (vestibulocochlear implant), CI (cochlear implant), VI (vestibular implant).

**Fig 4:**
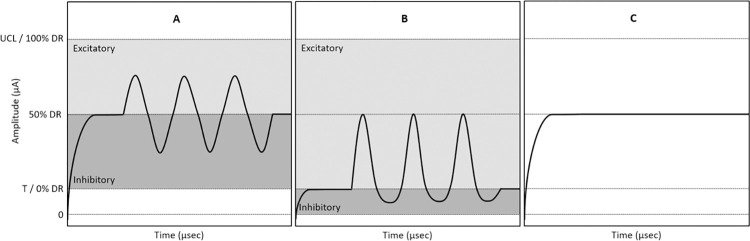
Three modes of stimulation by the vestibular implant. (A) Motion-modulated stimulation with baseline stimulation. (B) Motion-modulated stimulation with reduced baseline stimulation. (C) Baseline stimulation (no modulation). Baseline stimulation is given as a constant-amplitude electrical pustule signal in all three stimulation modes. Excitatory modulation is shown in light gray and inhibitory modulation is shown in dark gray.
