# Supplementary material for: The VertiGO! Trial protocol: A prospective, single-center, patient-blinded study to evaluate efficacy and safety of prolonged daily stimulation with a multichannel vestibulocochlear implant prototype in bilateral vestibulopathy patients
Source: PLoS One. 2024 Mar 28;19(3):e0301032. doi: 10.1371/journal.pone.0301032 (PMC10977751; doi:10.1371/journal.pone.0301032)
Supplement: S1 Appendix — (DOCX) [file pone.0301032.s001.docx]

**S1 Appendix. Model informed consent form**

Belonging to: The VertiGO! Trial

- I have read the information sheet. I was able to ask questions. My questions have been answered well enough. I had enough time to decide if I wanted to take part.
- I know that taking part is voluntary. I also know that at any time I can decide not to take part in the study. Or to stop taking part. I do not have to explain why.
- I give the investigator consent to inform my doctor that I am taking part in this study.
- I give consent to collect and use my data. The investigators only do this to answer the question of this study.
- I know that some people will be able to see all of my data to review the study. These people are mentioned in this information sheet. I give consent to let them see my data for this review.
- I know that my coded data will also be sent to countries outside the European Union where the privacy rules of the European Union do not apply. I consent to this.
- Please tick yes or no in the table below.

| I give consent to store my data to use for other research, as stated in the information sheet. | Yes ☐ | No☐ |
| --- | --- | --- |
|  |  |  |
| I give consent to ask me after this study if I want to participate in a follow-up study. | Yes ☐ | No☐ |
| I give consent to let me know after the study which treatment I received/in which group I was. | Yes ☐ | No☐ |

- I want to take part in this study.

My name is (subject): ………………………………..

Signature: ……………………… Date : __/__/__

-----------------------------------------------------------------------------------------------------------------

I declare that I have fully informed this subject about the study mentioned.

If any information becomes known during the study that could influence the subject's consent, I will let this subject know in good time.

Investigator name (or their representative): ........................

Signature:……………………… Date: __/__/__
