# Supplementary material for: The VertiGO! Trial protocol: A prospective, single-center, patient-blinded study to evaluate efficacy and safety of prolonged daily stimulation with a multichannel vestibulocochlear implant prototype in bilateral vestibulopathy patients
Source: PLoS One. 2024 Mar 28;19(3):e0301032. doi: 10.1371/journal.pone.0301032 (PMC10977751; doi:10.1371/journal.pone.0301032)
Supplement: S3 Appendix — (DOCX) [file pone.0301032.s003.docx]

**World Health Organization Trial Registration Data Set**

| **Data category** | **Information** |
| --- | --- |
| Primary registry and trial identifying number | ClinicalTrials.gov NCT04918745 |
| Date of registration in primary registry | April 28, 2021 |
| Secondary identifying numbers | METC20-087 ( Other Identifier: METC azM/UM ) |
| Source(s) of monetary or material support | Maastricht University Medical Center |
| Primary sponsor | Maastricht University Medical Center |
| Secondary sponsor(s) | MED-EL, Health Holland, Heinsius-Houbolt Fund |
| Contact for public queries | bernd.vermorken@mumc.nl |
| Contact for scientific queries | bernd.vermorken@mumc.nl |
| Public title | The VertiGO! Trial: a prospective, single-center, patient-blinded, three-period crossover study to evaluate efficacy and safety of prolonged daily stimulation with a multichannel vestibulocochlear implant prototype in bilateral vestibulopathy patients |
| Scientific title | The VertiGO! Trial: a prospective, single-center, patient-blinded, three-period crossover study to evaluate efficacy and safety of prolonged daily stimulation with a multichannel vestibulocochlear implant prototype in bilateral vestibulopathy patients |
| Countries of recruitment | Netherlands |
| Health condition(s) or problem(s) studied | Bilateral vestibulopathy |
| Intervention(s) | Device: Cochlear Vestibular Implant (CVI)  The Cochlear Vestibular Implant (CVI) is a modified cochlear implant (CI) which also incorporates a vestibular component (VI) in order to restore both hearing and vestibular function. Three vestibular stimulation algorithms will be compared in a randomized order in a crossover design. These stimulation algorithms are: A - Constant stimulation, without modulation B - Modulated stimulation with constant stimulation at 50% of the dynamic range C - Modulated stimulation with constant stimulation on a reduced level |
| Key inclusion and exclusion criteria | Inclusion criteria:   1. Chronic vestibular syndrome being presented by disabling symptoms of postural imbalance and/or impaired image stabilization (e.g. oscillopsia) 2. Reduced or absent bilateral VOR function based on at least one of the tests below meeting criteria A, with the other tests meeting criteria B:   Criteria A: Caloric response: Each side ≤6°/sec, vHIT gain: Bilateral horizontal SCC ≤ 0.6 AND Bilateral vertical SCC <0.7, Rotatory chair gain: ≤ 0.1 (0.1 Hz)  Criteria B: Caloric response: Each side <10°/sec, vHIT gain: 2 Bilateral SCC <0.7, Rotatory chair gain: ≤ 0.2 (0.1 Hz)   1. Onset of bilateral vestibular loss after the age of 2 2. Vestibular dysfunction from a peripheral origin or idiopathic BV 3. Patent vestibular end-organ (judged by CT) 4. Vestibular function and symptoms have not recovered beyond inclusion criteria within 6 months from onset of symptoms including a 3 month rehabilitation program off vestibular suppressant medications 5. Meeting CI-candidacy in ear to implant with CVI 6. Agreed to receive a MED-EL CVI implant with MED-EL sound processor 7. Capacitated adults ≥ 18 years 8. Proficient speaker of the Dutch language 9. No contra-indications for CVI surgery 10. Active participation in the trial related procedures such as regular testing, the VI fitting period, the baseline testing day and three weeks of intensive VI rehabilitation and testing in the study center (MUMC+) including an exercise regimen 11. Agreed not to swim or to use or operate vehicles, heavy machinery, powered tools or other devices that could pose a threat to the participant, to others, or to property throughout the period of VI activation and until at least 1 day after VI deactivation   Remark: Patients who qualify to receive a regular CI as part of standard clinical care will have a preferential position to be included in the trial.  Exclusion Criteria:   1. Signs of central vestibular/cochlear dysfunction or structural vestibular/cochlear nerve pathology (judged by physical examination / MRI) 2. Clear signs of structural nerve pathology or indications of improperly functioning vestibular/cochlear nerves 3. Requirement for electric-acoustic activation of the CI part (e.g. "hybrid" processor) prior to completion of the prolonged VI stimulation period 4. Having received a cochlear implant earlier on the side to implant (e.g. explantation/reimplantation) 5. Having received a cochlear implant from another brand than MED-EL in the other ear (bilateral implantation with different brands is not supported) 6. Unwillingness to stop the use of antihistamines which might suppress VOR responses (e.g. cinnarizine) in the period of 1 month before until after each measurement point. 7. Pre-lingual onset of bilateral profound deafness (< 4 years of age) 8. Active participation in another prospective clinical trial 9. Pregnancy or having plans to become pregnant at the time of imaging or during the VI trial 10. Orthopedic, ocular, neurologic or other non-vestibular pathologic conditions of sufficient severity to confound vestibular function tests used in the study 11. Current psychological or psychiatric disorders that could significantly interfere with the use or evaluation of VI stimulation 12. Physical or non-physical contraindications for MRI or CT imaging prior to surgery 13. Making chronic use of psychiatric medication which suppresses VOR responses (e.g. SSRI's, benzodiazepines) 14. Significant dental problems which prohibit the stable use of a 'bite bar' (used as calibration reference for the gyroscope functionality of the CVI) 15. Any medical condition, judged by the research team, that is likely to interfere with a study candidate's participation in the study |
| Study type | Interventional Allocation: randomized Masking: single blind Primary purpose: treatment |
| Date of first enrolment | July 2021 |
| Target sample size | Minimum of 8, maximum of 13 |
| Recruitment status | Recruiting |
| Primary outcome(s) | Safety and efficacy of stimulation with a vestibulocochlear implant Fundamental insights in comparing three stimulation modes with a vestibulocochlear implant |
| Key secondary outcomes | Bidirectional CI and VI interactions Auditory performance with a VCI Long-term follow-up of a VCI  Patient reported outcome measures / patient perspectives of acute and prolonged VCI stimulation  Imaging and electrode placement  Proof of Concept of VI fitting Proof of Concept of a VI rehabilitation program  Evaluating different stimulation parameters (modulations, transfer functions) |
