# Supplementary material for: The VertiGO! Trial protocol: A prospective, single-center, patient-blinded study to evaluate efficacy and safety of prolonged daily stimulation with a multichannel vestibulocochlear implant prototype in bilateral vestibulopathy patients
Source: PLoS One. 2024 Mar 28;19(3):e0301032. doi: 10.1371/journal.pone.0301032 (PMC10977751; doi:10.1371/journal.pone.0301032)
Supplement: S1 Protocol — (PDF) [file pone.0301032.s004.pdf]

RESEARCH PROTOCOL

October 2022

VertiGO! - Get up and GO! with the vestibular implant

**Protocol ID**	ABR 73492
**Short title**	VertiGO!
**Version**	6
**Date**	21-10-2022
**Coordinating investigator**	Dr. R. van de Berg, MD, ENT-physician, Head of Vestibular Department<br>Department of ENT<br>Maastricht University Medical Center, The Netherlands<br>raymond.vande.berg@mumc.nl
**Principal investigators**	Dr. R. van de Berg, MD, ENT-physician, Head of Vestibular Department<br>Prof. Dr. Herman Kingma, Vestibular Research Professor Department of ENT,<br>Maastricht University Medical Center, The Netherlands<br>raymond.vande.berg@mumc.nl<br>herman.kingma@mumc.nl
**Researcher**	Drs. Bernd Vermorken, ENT-physician in training<br>Department of ENT,<br>Maastricht University Medical Center, The Netherlands<br>bernd.vermorken@mumc.nl
**Reseacher**	Drs. Benjamin Volpe, ENT-physician in training<br>Department of ENT,<br>Maastricht University Medical Center, The Netherlands<br>benjamin.volpe@mumc.nl
**Researcher**	Ir. Stan van Boxel, Clinical physicist, audiologist in training<br>Department of ENT<br>Maastricht University Medical Center, The Netherlands<br>stan.van.boxel@mumc.nl
**Researcher**	Drs. Elke Loos, ENT-physician<br>Department of ENT,<br>Maastricht University Medical Center, The Netherlands<br>elke.loos@mumc.nl

**Researcher**	Drs. M. van Hoof, ENT-physician in training<br>Department of ENT,<br>Maastricht University Medical Center, The Netherlands<br>marc. hoofvan@mumc.nl
**Researcher**	Dr. E. M. J. Devocht, Master of Audiology<br>Department of ENT,<br>Maastricht University Medical Center, The Netherlands<br>elke.devocht@mumc.nl
**Sponsor**	azM<br>P. Debyelaan 25<br>6229 HX Maastricht, The Netherlands
**Subsidizing parties**	ZonMW/Health Holland<br>Heinsius Houbolt Fonds<br>MED-EL
**Independent expert**	Dr. Laura Baijens, MD, ENT-physician/surgeon<br>Department of ENT,<br>Maastricht University Medical Center, The Netherlands<br>laura.baijens@mumc.nl
**Statistician**	A. Miranda L. Janssen, MSc, Assistant Statistical Analist<br>Department Methodology & Statistics<br>Maastricht University, The Netherlands<br>miranda.janssen@maastrichtuniversity.nl

PROTOCOL SIGNATURE SHEET

Name	Signature	Date
**Sponsor or legal representative:**<br>Maastricht UMC+<br>P. Debyelaan 25<br>6229 HX Maastricht<br>The Netherlands<br>Represented by:<br>*Prof. dr. B. Kremer, ENT-physician/surgeon*		
**Principal Investigator:**<br>*Dr. Raymond van de Berg, MD, ENT-physician, Head of Vestibular Department*<br>Department of ENT,<br>Maastricht University Medical Center, The Netherlands		

TABLE OF CONTENTS

1. INTRODUCTION AND RATIONALE 15
   1.1. The vestibular system 15
   1.2. Vestibular function 16
   1.3. Vestibulopathy 16
   1.4. The vestibular implant 17
2. OBJECTIVE 20
   2.1. Primary objectives 20
   2.2. Secondary objectives 20
3. STUDY DESIGN 21
   3.1. Study design rationale 21
   3.2. Study planning 21
4. STUDY POPULATION 26
   4.1. Population (base) 26
   4.2. Inclusion criteria 26
   4.3. Exclusion criteria 27
   4.4. Sample size calculation 28
5. TREATMENT OF SUBJECTS 29
   5.1. Investigational product/treatment 29
   5.2. Use of co-intervention (if applicable) 32
   5.3. Escape medication (if applicable) 32
6. INVESTIGATIONAL PRODUCT 33
   6.1. Name and description of investigational product 33
   6.2. Summary of findings from non-clinical studies 33
   6.3. Summary of findings from clinical studies 33
   6.4. Summary of known and potential risks and benefits 33
   6.5. Description and justification of route of administration and dosage 36
   6.6. Dosages, dosage modifications and method of administration 36
   6.7. Preparation and labelling of Investigational Medicinal Product 36
   6.8. Drug accountability 37
7. NON-INVESTIGATIONAL PRODUCT 38
   7.1. Name and description of non-investigational product(s) 38
   7.2. Summary of findings from non-clinical studies 38
   7.3. Summary of findings from clinical studies 38

7.4. Summary of known and potential risks and benefits 38
7.5. Description and justification of route of administration and dosage 38
7.6. Dosages, dosage modifications and method of administration 38
7.7. Preparation and labelling of Non Investigational Medicinal Product 38
7.8. Drug accountability 38
8. METHODS 39
8.1. Study parameters/endpoints 39
8.2. Randomization, blinding and treatment allocation 41
8.3. Study procedures 41
8.4. Withdrawal of individual subjects 54
8.5. Replacement of individual subjects after withdrawal 55
8.6. Follow-up of subjects withdrawn from treatment 55
8.7. Premature termination of the study 55
9. SAFETY REPORTING 57
9.1. Temporary halt for reasons of subject safety 57
9.2. AEs, SAEs and SUSARs 57
9.3. Annual safety report 58
9.4. Follow-up of adverse events 58
9.5. Data Safety Monitoring Board (DSMB) 58
10. STATISTICAL ANALYSIS 59
10.1. Primary study parameter(s) 59
10.2. Secondary study parameter(s) 59
10.3. Other study parameters 60
10.4. Interim analysis 60
11. ETHICAL CONSIDERATIONS 61
11.1. Regulation statement 61
11.2. Recruitment and consent 61
11.3. Objection by minors or incapacitated subjects 61
11.4. Benefits and risks assessment, group relatedness 61
11.5. Compensation for injury 62
11.6. Incentives 62
12. ADMINISTRATIVE ASPECTS, MONITORING AND PUBLICATION 63
12.1. Handling and storage of data and documents 63
12.2. Monitoring and Quality Assurance 63

12.3. Amendments 63
12.4. Annual progress report 64
12.5. Temporary halt and (prematurely) end of study report 64
12.6. Public disclosure and publication policy 64
13. STRUCTURED RISK ANALYSIS 65
13.1. Potential issues of concern 65
13.2. Synthesis 69
14. REFERENCES 71

LIST OF ABBREVIATIONS AND RELEVANT DEFINITIONS

(S)AE	(Serious) Adverse Event
3D HIT	3 Dimensional Head Impulse Test
ABR	General Assessment and Registration form (ABR form), the application form that is required for submission to the accredited Ethics Committee; in Dutch: Algemeen Beoordelings- en Registratieformulier (ABR-formulier)
AE	Adverse Event
AMP	Audio-Motion Processor
AR	Adverse Reaction
ART	Auditory nerve Response Telemetry
azM	Academic Hospital Maastricht
BV	Bilateral Vestibulopathy
CA	Competent Authority
CAREN	Computer Assisted Rehabilitation ENvironment
CBCT	Cone Beam Computed Tomography
CCMO	Central Committee on Research Involving Human Subjects; in Dutch: Centrale Commissie Mensgebonden Onderzoek
CI	Cochlear Implant
CNC	Consonant-Nucleus-Consonant
CT	Computed Tomography
CTCM	Clinical Trial Center Maastricht
CV	Curriculum Vitae
cVEMP	cervical Vestibular Evoked Myogenic Potential
CVI	Cochlear-Vestibular Implant
DHI	Dizziness Handicap Inventory
DIN	Digits-In-Noise
DR	Dynamic Range
DSMB	Data Safety Monitoring Board
DVA	Dynamic Visual Acuity
ECAP	electrically evoked Compound Action Potential
eABR	electrically evoked Auditory Brainstem Response

**eVBR**	**Electrically evoked Vestibular Brainstem Response**
**EMG**	**ElectroMyoGraphy**
**ENG**	**Electro-NystagmoGraphy**
**EQ5D-5L**	**EuroQol five-Dimensional questionnaire**
**EU**	**European Union**
**EudraCT**	**European drug regulatory affairs Clinical Trials**
**FES-I**	**Falls Efficacy Scale - International**
**fHIT**	**functional Head Impulse Test**
**GCP**	**Good Clinical Practice**
**GDPR**	**General Data Protection Regulation; in Dutch: Algemene Verordening Gegevensbescherming (AVG)**
**HADS**	**Hospital Anxiety and Depression Scale**
**HUI-3**	**Health Utility Index-3**
**IB**	**Investigator's Brochure**
**IC**	**Informed Consent**
**ICECAP-A**	**ICEpop CAPability measure for Adults questionnaire**
**IMP**	**Investigational Medicinal Product**
**IMPD**	**Investigational Medicinal Product Dossier**
**LARP**	**Left-Anterior-Right-Posterior**
**LIST**	**Leuven Intelligibility Sentence Test**
**logMAR**	**logarithm of the Minimum Angle of Resolution**
**METC**	**Medical research ethics committee (MREC); in Dutch: medisch-ethische toetsingscommissie (METC)**
**Mini-BESTest**	**Mini-Balance Evaluation Systems Test**
**MRI**	**Magnetic Resonance Imaging**
**MRV**	**Mean Rectified Voltage**
**MUMC+**	**Maastricht University Medical Center**
**OSQ**	**Oscillopsia Severity Questionnaire**
**oVEMP**	**Ocular Vestibular Evoked Myogenic Potential**
**PSFS**	**Patient Specific Functional Scale**
**QALY**	**Quality Adjusted Life Year**

**QMS**	**Quality Management System**
**QoL**	**Quality of Life**
**RALP**	**Right-Anterior-Left-Posterior**
**SCC**	**Semi Circular Canal**
**SNHL**	**Sensory Neural Hearing Loss**
**SPC**	**Summary of Product Characteristics; in Dutch: officiële productinformatie IB1-tekst**
**SPIN**	**SPeech In Noise**
**Sponsor**	**The sponsor is the party that commissions the organization or performance of the research, for example a pharmaceutical company, academic hospital, scientific organization or investigator. A party that provides funding for a study but does not commission it is not regarded as the sponsor, but referred to as a subsidizing party.**
**SRT**	**Speech-Reception-Threshold**
**SSQ-12**	**Speech-Spatial-Qualities of hearing scale**
**SUSAR**	**Suspected Unexpected Serious Adverse Reaction**
**T**	**Threshold**
**TQ**	**Tinnitus Questionnaire**
**UAVG**	**Dutch Act on Implementation of the General Data Protection Regulation; in Dutch: Uitvoeringswet AVG**
**UCL**	**Upper Comfortable Limit**
**UZA**	**Antwerp University Hospital**
**VA**	**Visual Acuity**
**vHIT**	**video Head Impulse Test**
**VI**	**Vestibular Implant**
**VOG**	**Video-OculoGraphy**
**VOR**	**Vestibulo-Ocular Reflex**
**VSR**	**Vestibulo-Spinal Reflexes**
**WMO**	**Medical Research Involving Human Subjects Act; in Dutch: Wet Medisch-wetenschappelijk Onderzoek met Mensen**

SUMMARY

Rationale: The vestibular sensory organ is essential for balance and image stabilization. It drives the eyes to keep the image of the environment stable. Severe function loss of both vestibular organs is referred to as bilateral vestibulopathy (BV). Patients with BV present themselves with serious day-to-day disabilities such as strong balance disturbances, higher risk of falling, visual symptoms (oscillopsia) and a loss of autonomy. It has been conservatively estimated that more than 200.000 people in Europe and 2.1 million people worldwide are affected. Up until now no effective treatment is available for these patients. In the past years experimental electric stimulation of the vestibular nerve in humans by means of a vestibular implant (VI) has shown to be able to partly restore balance and gaze functionality in test situations. After showing feasibility of vestibular stimulation in humans in previous trials (NL36777.068.11, NL31405.068.10), the current step in this trial will be to show that prolonged daily stimulation is effective and safe.

Objectives:

1. Primary
    a. To investigate the efficacy of prolonged daily VI stimulation
    b. To investigate the safety of prolonged daily VI stimulation
    c. To investigate the efficacy of three different types of stimulation:

	Baseline stimulation	Motion-modulation
1)	On	Off
2)	On	On
3)	Reduced	On

2. Secondary
    a. To develop a rehabilitation program for VI recipients
    b. To evaluate the interaction between vestibular and cochlear nerve stimulation
    c. To evaluate acute VI performance across several years

Study design: Controlled clinical trial with a randomized single-blind cross-over design

Study population: Eight to thirteen patients who have (1) disabling symptoms of BV and (2) severe sensorineural hearing loss (SNHL) in at least the ear to be implanted.

Intervention: To evaluate combined prolonged stimulation of both the vestibular organ and the cochlea, subjects will be implanted with a cochlear-vestibular implant (CVI). This extended CI also incorporates a vestibular component (VI) in order to restore both hearing and vestibular function. Once the prosthesis is implanted, the patient will pay scheduled visits to our clinic. Hearing rehabilitation with the CI will follow the standard clinical protocol. Evaluation of the CVI functionality will include a CBCT scan 1 week post-op, 1 hour extra testing at the end of the CI rehabilitation period, a VI fitting period (4 days), a day of baseline testing, 3 periods of 4 days prolonged VI stimulation and tests conducted during regular CI follow-up visits until 5 years after implantation, including 3 CBCT scans (12 hours extra during standard visits). An overview of the trial schedule is given in figure 1. More detailed overviews can be found in chapter 3. During the fitting period and the prolonged stimulation period the patients will be staying in a hotel next to the hospital. During the prolonged stimulation period, subjects will follow a vestibular rehabilitation program and the outcomes will be evaluated using a set of vestibular tests (including video-oculography (VOG), perception tests and test of gait), auditory tests, semi-structured qualitative interviews and questionnaires.

Outcome measures:

1. *Primary:* The main outcome measure is image stabilization based on dynamic visual acuity (DVA). Complementary to the main outcome measure, a set of auxiliary outcome measures are included to assess all functional aspects of combined vestibular and cochlear stimulation. These include a gait analysis to assess locomotion, video-oculography (VOG) to assess gain of stimulation, perception of movement to assess stimulation perception and hearing tests to assess CI functionality.
2. *Secondary:* Subjective outcomes based on questionnaires and semi-structured qualitative interviews.

Endpoints per objective (see above):

1. Primary objectives
   a. Primary and secondary outcome measures will be compared between conditions with VI stimulation (VI-ON) versus without stimulation (VI-OFF). The primary and secondary outcome measures will also be used to monitor the effect of prolonged stimulation by comparing outcomes at different time points during the prolonged stimulation period.

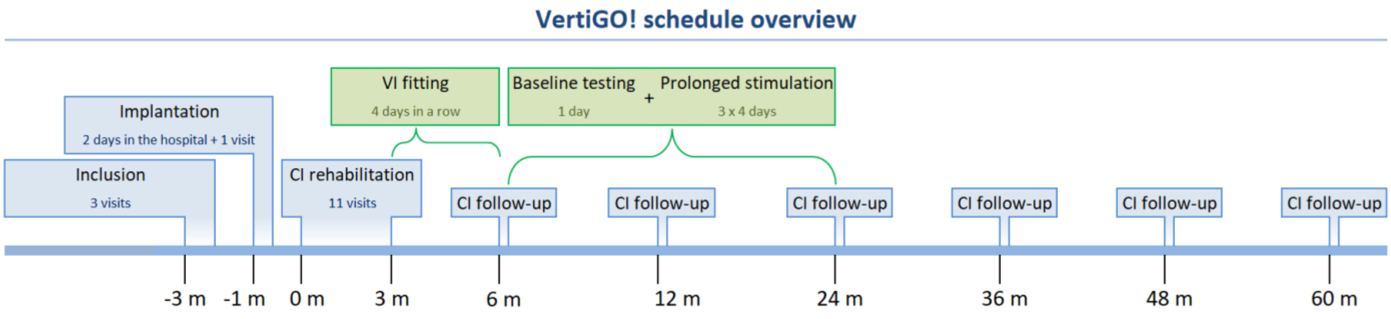

Figure 1: General overview of the VertiGO! trial schedule

   b. *Amount of (S)AE's or other undesired side effects (e.g. psychological burden) derived from secondary outcome measures in relation to prolonged daily VI stimulation.*
   c. Primary and secondary outcome measures will be compared between the different stimulation algorithms used during the different prolonged stimulation periods.
2. Secondary objectives
   a. Tailor made rehabilitation programs
   b. Primary and secondary outcome measures will be used to investigate the interaction between vestibular stimulation and CI functionality
   c. A subset of primary and secondary outcomes during long-term clinical follow-up up to 5 years

Nature and extent of the burden and risks associated with participation, benefit and group relatedness:

The main burden associated with participation is significant time investment. Patients will undergo both CI inclusion testing (2 visits) and VI inclusion testing (1 visit) and be admitted for surgery (2 days). After surgery, patients will visit the hospital for the regular CI visits and for trial-specific visits. The trial specific visits include a VI fitting period (4 days), baseline testing (1 day) and the prolonged stimulation period (3*4 days). During a number of the regular CI visits extra tests will be conducted specifically for the trial (see table 1, chapter 4 of the research protocol). During the fitting period and the prolonged stimulation period, patients will be staying in a hotel next to the hospital to reduce the burden of travel on the patients. During the visits, rehabilitation exercises, vestibular tests, hearing tests and interviews will be executed. All of these activities are associated with a low physical and psychological burden. Personal limits of patients will explicitly be taken into account.

The most substantial risks involved with receiving a CVI is damage to the neural structures within the cochlea and the vestibular system. Therefore, we apply stringent inclusion criteria and will only implant patients with disabling symptoms of BV and severe SNHL in the ear to be implanted, to minimize the amount of potential damage that can be done. Alongside this, outside of the research setting, the CVI implant will function as a regular CI, restoring hearing in the ear to be implanted and compensating for the loss in natural hearing potentially occurring by inserting the electrodes into the inner ear. Other risks associated with implantation of the CVI are estimated to be minimal based on previous CVI implantations performed in our clinic (NL36777.068.11, NL31405.068.10) and similar to risks associated with implanting and using a regular CI.

Risks of vestibular stimulation via the CVI include dizziness, nausea and a risk of falling. The risks of dizziness and nausea will most likely subside as the patient gets more used to vestibular stimulation, and can always be negated by stopping the vestibular stimulation. The risk of falling, alongside any unforeseen risks, is minimalized by taking care and time to let the patient get used to vestibular stimulation and by only applying vestibular stimulation while the patient is in the hospital under supervision by a member of the research team. While the patient receives vestibular stimulation via the CVI, the performance of the CI-part of the implant might (temporarily) be reduced.

The benefit for the patient is that via the CI functionality of the CVI their hearing will most likely improve, analogous to a regular CI. The VI functionality of the CVI can only be used within a research setting, and will therefore not result in a direct patient benefit outside of trial related activities under supervision in the hospital.

Concerning group relatedness, BV represents a major handicap with strong balance disturbances, higher risk of falling, visual symptoms (oscillopsia) and a loss of autonomy. There is no therapeutic strategy at the moment. Development of a vestibular prosthesis could help this group of patients to have a better quality of life. With this study we hope to answer crucial research questions (see chapter 1.4 of the research protocol) and bring essential knowledge towards the development and refining of a vestibular prosthesis. This trial is part of a larger plan to develop the concept of a VI which can be adopted into clinics. As described in chapter 1.4, substantial research has been conducted with VI stimulation in the past. This trial serves as the next step in this process. If this trial succeeds in proving the safety and efficacy of prolonged vestibular stimulation, future trials with a larger patient cohort are in the pipeline to build on the knowledge gathered in this trial.

1. INTRODUCTION AND RATIONALE

1.1. The vestibular system

The vestibular system is a set of sensory organs which detect angular and linear acceleration of the head. Housed within the inner ear, it is composed of 3 semicircular canals (SCCs) and 2 otolith organs, with a duplicate system on the contralateral side. The vestibular system, synergically with the optokinetic, somatosensory and proprioceptive systems, plays a key role in spatial orientation, postural control and gaze stabilization [1].

1.1.1. *Semicircular canals: angular rotations*

SCCs are sensitive to angular acceleration. The three SCCs are positioned orthogonally from each other (lateral, posterior and anterior). Each SCC has an ampullated limb at its end which contains its neurosensory structure. Referred to as the ampulla, it hosts hair cells which are covered by a gelatinous cupula.

By angular accelerations in the plane of the SCC, i.e. head rotations, the cupula deflection due to inertia of the endolymph fluid inside the SCC induces changes in the hair cells receptor potential. Angular head accelerations towards a specific canal's rotational axis induce depolarization of the hair cells and increase the firing rate of the vestibular nerve fibers. Angular head accelerations away from a specific canal's rotational axis induce hyperpolarization of the hair cells and decrease the firing rate of the vestibular nerve fibers. Potential and firing frequency changes are proportional to the acceleration, but saturate at various angular accelerations (varies from hair cell to hair cell).

The spontaneous firing frequency in the nerve fibers in rest is about 90 spikes/sec [2,3]. This spontaneous electric activity frequency can decrease down to 0 spikes/sec and increase up to 400 spikes/sec [2,3]. This results in an asymmetric sensitivity of hair cells and canals. In healthy subjects this functional asymmetry is silent because vestibular responses are generated by canals of both inner ears. However, this does become clinically relevant in unilateral vestibular lesion.

1.1.2. *Otolith organs: linear accelerations*

The otolith organs, the saccule and the utricle, both have a macula that contains the neurosensory structure. It is composed of hair cells covered by a gelatinous otolithic membrane and otoconiae. They respond to linear acceleration, such as the pull of gravity [2] and are especially important for postural control.

1.2. Vestibular function

Electric activity modulations of the canals' ampullae and otolith organs' maculae allow the generation of vestibulo-spinal reflexes (VSR), for postural control, and vestibulo-ocular reflexes (VOR), for gaze stabilization. The VOR generated by ampullary organs results in a synchronous eye deviation opposite to the head movement direction which can be registered by electro-nystagmography (ENG) or VOG. Over the last 50 years, both have been routinely used to assess the vestibular system for horizontal stimulations. It has only been recently that new clinically available tests have been developed allowing investigation of vertical canals and otolith organs as well [4–6].

1.3. Vestibulopathy

The importance of the vestibular system's role in body posture, body motion control and perception is often underestimated. Its importance generally becomes clear in motion sickness and after vestibular system failure. The etiology of vestibular loss can be ototoxic, infectious, traumatic, autoimmune, congenital or idiopathic.

Vestibular loss can be divided into two subcategories: unilateral and bilateral loss. Unilateral vestibular loss usually doesn't lead to long term severe symptoms. Plasticity of the central nervous system namely allows efficient compensatory mechanisms to be enforced. In this way unilateral vestibular loss can partly be compensated for, although specific deficits remain [7]. Bilateral vestibular loss, referred to as BV, does often lead to permanent problems.

Patients with BV experience imbalance, disorientation, vertigo and discomfort during all types of movements, leading to impairments and restrictions in functioning during most day-to-day activities. It has been conservatively estimated that >200.000 people in Europe and >2.1 million people worldwide are affected [8]. The prevalence is likely to be underestimated [9]. In absence of treatment options, there is less awareness, inclination and incentive for a formal diagnosis. Other than the obvious burden of disease, BV is associated with reduced societal participation, reduced quality of life (QoL) and a 31x higher risk of falling [10].

Although progress is being made with devices specifically targeting certain deficits caused by BV, such as sensory substitution devices to help impaired balance [11], or devices which apply noisy electrical stimulation to enhance residual function [12], no treatment option to reestablish the full functionality of the vestibular organs exist today. In response to this absence, the concept of a vestibular prosthesis, in analogy with a cochlear prosthesis, is an ongoing and important field of research.

1.4. The vestibular implant

1.4.1. *The concept of a vestibular prosthesis*

The VI is a bio-electronic prosthesis that could restore vestibular function, much in the same way a CI restores hearing. It aims to provide the central nervous system with information about spatial orientation and movement, thereby replacing the function of the vestibular sensory organs. A sensor containing accelerometers and gyroscopes is applied to the patients' head, where it measures head angular velocity and linear acceleration in all axes of movement. The measured signals are then transduced into electric signals, which are delivered to the vestibular nerves by implanted electrodes [13].

1.4.2. *Development of the VI*

The Geneva-Maastricht group is one of a few groups worldwide that is working on the concept of electrical vestibular stimulation and the first group to implant a VI in humans. The point of action has been identified and it was shown that vestibular nerves can be successfully stimulated electrically. The current knowledge on VI stimulation results from our research with the biggest VI-patient cohort in the world (13 patients), of which 5 patients were implanted in Maastricht (NL36777.068.11).

It started in 2007 with implanting a CVI with a single vestibular electrode [13]. After development of additional surgical techniques [14–16], and successful intra-operative vestibular stimulation [17], the first implants with multiple vestibular electrodes were implanted in 2012. The possibility of hearing preservation and the feasibility of additional surgical tools were presented [18,19]. It was shown that the CVI is a safe and effective way of stimulating the vestibular system, even in a heterogeneous patient population [13,20–24]. A relevant functional benefit was shown by improving vision during walking [25]. Therefore, it can be stated that substantial, promising results have been published and replicated by us and just a few others [13,16,18,20–24,26].

Alongside this, the Geneva-Maastricht group extensively described the patient population [27], proposed the diagnostic work-up for this disease [9,27] and was involved in the development of the diagnostic criteria for BV [28]. Qualitative interviews with BV patients have been conducted to obtain symptoms, cost-utility analyses and patient expectations of a VI. Manuscripts regarding these topics have been published or are currently in preparation [27,29,30].

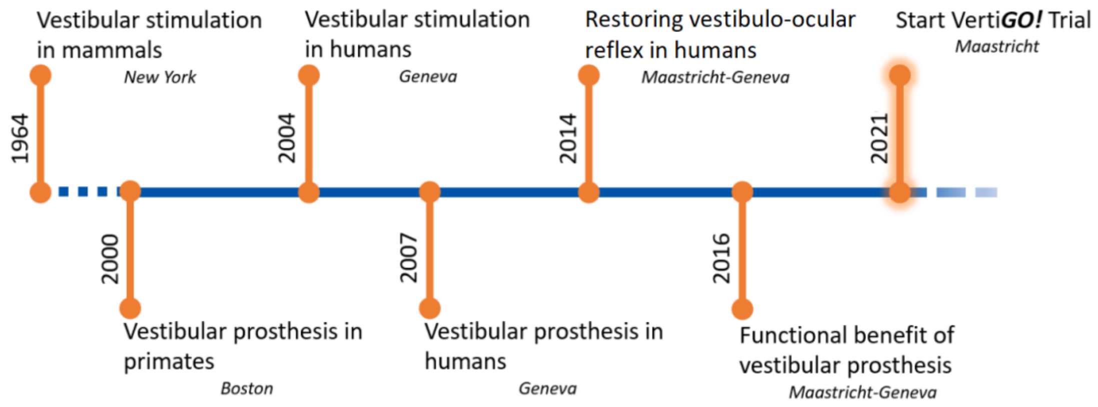

Figure 3. History of published VI-research

1.4.3. *Prolonged stimulation*

The above-mentioned results indicate the feasibility of a VI as a therapeutic option for (at least) BV-patients in the near future. Most previous studies published up until now report only short-term or intermittent use of a vestibular prosthesis [13,16,18,20–22,24–26,31]. Recently a few research groups started to publicize papers on the long term effects of vestibular stimulation [32–34]. However, as the authors of these papers mention themselves as well, the constraints in their study designs prevent them from generalizing their results to a larger patient population or creating more general statements of VOR response over time. Therefore, there is a need to investigate safety and efficacy of chronic daily stimulation in a more rigorous trial setting.

Despite the restriction in studies done so far, the results do point towards the feasibility of long term stimulation, indicated by stable VOR response over time approximately aligned with any specified head rotational axis. Animal studies [35] have shown that most of the adaption to misalignment that might be present between the actual head rotation and the stimulated VOR response gets compensated for during the first week of stimulation. Up until now this process has not been characterized in humans. Alongside this process it is likely that other adaptation processes would also occur during the first weeks of stimulation, potentially influencing the stimulation efficiency and induced responses. For other implants based on neurostimulation like for example the CI, the response to stimulation (e.g. dynamic range) changes substantially during the first weeks of stimulation and it takes several months before it plateaus at a more stable level [36]. This trial would allow for the evaluation of these processes during the first week of stimulation, giving valuable insights into the interaction between the VI and the human body, essential for the further development of the concept of electrical vestibular stimulation.

1.4.4. *Stimulation algorithm*

During this trial three types of stimulation algorithms will be compared: (1) baseline stimulation without motion modulation, (2) baseline stimulation with motion modulation, and (3) reduced baseline stimulation with motion modulation. Previous experiments performed by our group have mainly utilized a stimulation algorithm based on angular head movement, since this type of stimulation is most similar to the movement which is perceived by the SCCs (location of the implanted electrodes).We aim to compare two motion-modulated (angular head movement) stimulation algorithms in this trial. By making use of baseline stimulation (e.g. constant stimulation at 50% of the dynamic range), it is possible to down- and upmodulate the given electrical stimulation depending on head-movement. This way, the electrical current is able to code for movements in both directions (excitation and inhibition) to the same extend. Alternatively, reducing the baseline stimulation (e.g. lower than 50% of the dynamic range) increases the “room” for modulation in one direction (excitation) while lowering it in the other direction (inhibition). This stimulation algorithm is expected to boost the excitation response, possibly resulting in stronger compensatory eye-movements in one direction. To serve as a control for both modulated stimulation algorithms, an algorithm consisting of un-modulated stimulation is also included in the trial. Previous research has shown the positive effects of constant vestibular nerve stimulation without head movement based signal modulation [37]. The two main theories behind the effectiveness of un-modulated stimulation are the enhancement of the residual vestibular function (due to stochastic resonance) and the enhancement of the function of other, remote senses. A comparison between the efficacy of prolonged

constant and modulated stimulation has not yet been reported in literature, even though this is a vital part of the substantiation for stimulation of the vestibular system with modulated stimulation based on head movement.

1.4.5. *Rehabilitation*

Physiotherapeutic rehabilitation is an integral part of current therapy for patients suffering from vestibular loss [38,39]. The goals of this rehabilitation are to train a patient in using the remaining function of their vestibular system, to increase their use of other sensory systems and to teach compensatory behavior. Alongside this, observing results from the field of CI rehabilitation [40], there are clear indications to the efficacy of specialized rehabilitation programs being used in combination with implants based on neurostimulation. Therefore, we assume that a rehabilitation program analogous to a normal BV rehabilitation will also be beneficial in combination with the VI, improving its efficacy and usability. This VI-specific rehabilitation program under development here will focus on challenging the patient to make use of the VI while also training the patient to effectively interpret the information that is supplied. A rehabilitation program will potentially increase the therapeutic effect of the VI in this trial, besides giving proof-of-concept for a VI rehabilitation program and laying the ground work for the development of such a program which can be used in future VI-related research.

1.4.6. *Hearing*

Since the intended use of the CVI is both as a VI and a CI, restoring vestibular function and hearing at the same time, it is necessary to assess both functions of the implant working simultaneously. Phillips et al.[41] have shown that when stimulating the inner ear with a similarly designed implant to the CVI there is interaction between the electrodes placed in the vestibular organ and the cochlea. When vestibular and cochlear electrodes were stimulated simultaneously it could in some situations cause significant and potentially clinically relevant changes in electrically evoked slow-phase eye velocity, loudness percept and pitch percept. Likely caused by the spread of current, these results indicate the necessity to evaluate the interaction between VI and CI stimulation. By evaluating CI and VI performance when the CVI is used in a clinically relevant environment, these interactions can be investigated. The patients will be informed that there is a chance that the CI performance of the CVI will (temporarily) be reduced while the VI-part is active, since this can result in a burden for the patient. While optimizing the VI stimulation response during the VI fitting, the goal is to minimize the interaction between CI and VI stimulation and therefore minimize this burden.

2. OBJECTIVE

The primary objective of the VertiGO! trial is to assess the feasibility of restoring vestibular function by making use of prolonged VI stimulation. This will be done using a combined VI and CI device: The CVI. To achieve this objective, patients will use the wearable CVI device during three weeks within the safety of a hospital environment. This trial will serve as a proof-of-concept for restoring vestibular function to patients with BV, an as-of-yet untreatable disorder causing severe impairment and discomfort. The aims of this trial are to investigate efficacy and safety of prolonged vestibular stimulation, to identify the influence of different stimulation algorithms, to assess the feasibility of the combined VI/CI device, and to further build on the fundamental knowledge of vestibular organ stimulation while also taking into account the patient perspective.

2.1. Primary objectives

a. *To investigate the efficacy of prolonged daily VI stimulation:*
   It is expected that patients will benefit from modulated electrical stimulation of the SCC afferent nerves based on head movement information. Alongside this, any change in the stimulation effect will be monitored by repeated measurements during the stimulation period.
b. *To investigate the safety of prolonged daily VI stimulation:*
   It is expected that it will be safe to offer chronic vestibular stimulation to patients.
c. *To investigate the efficacy of three different types of stimulation: (1) motion-modulated stimulation with baseline stimulation, (2) motion-modulated stimulation with reduced baseline stimulation, and (3) baseline stimulation (no modulation):*
   It is expected that the modulated stimulation algorithms based on head movement are more effective at restoring VOR and VSR functions compared to unmodulated stimulation. It is also expected that motion modulation with a reduced baseline will increase VOR even further.
   It is expected that the stimulation algorithms based on head movement will be most effective at restoring all aspects of vestibular function.

2.2. Secondary objectives

a. *To develop a rehabilitation program for VI recipients*
   It is expected that patients will benefit from physiotherapeutic rehabilitation during prolonged VI stimulation by improving its functional outcome.
b. To evaluate the interaction between vestibular and cochlear nerve stimulation
   It is expected that there will be interaction between the stimulation of the vestibular organ and the cochlea. The extent and clinical relevance will be characterized.
c. *To evaluate acute VI performance across several years*
   It is expected that the CVI will induce stable VOR responses during acute stimulation over time. The clinical follow-up of the implanted patients will bring new insights for future research.

3. STUDY DESIGN

Study design: Randomized single-blind cross-over controlled clinical trial

3.1. Study design rationale

The study will be conducted with a cross-over design. Previous VI research has shown large variations between patients. For accurate assessments of the efficacy of different stimulation algorithms a within patient comparison is most suitable. Carry-over of stimulation effects are expected to be minimal based on previous experience with VI patients which showed repeatable results of acute stimulation with as little as one day of wash-out in between. Within this study design, the resting period between the different stimulation periods is chosen to be three days to decrease the burden on the patient and to further decrease the chance of carry-over.

Previous experience with vestibular stimulation has shown that the difference between stimulation on or off will be clearly noticeable by both the patients and the examiners. Therefore blinding the patient on whether or not they receive stimulation is unwarranted. To reduce further patient-induced bias, the patients will be blinded as to which stimulation algorithm is used in which week. Effort will be made to keep the bias induced by the researchers who conduct the examinations and the data analysis to a minimum. However, the current study set-up in combination with the current capabilities of the research device and fitting software do not allow for a fully double-blinded trial.

3.2. Study planning

The set of tables below describe the overall planning of the trial and the detailed planning of the different testing periods. An approximation for the amount of time required is given for the overall planning,

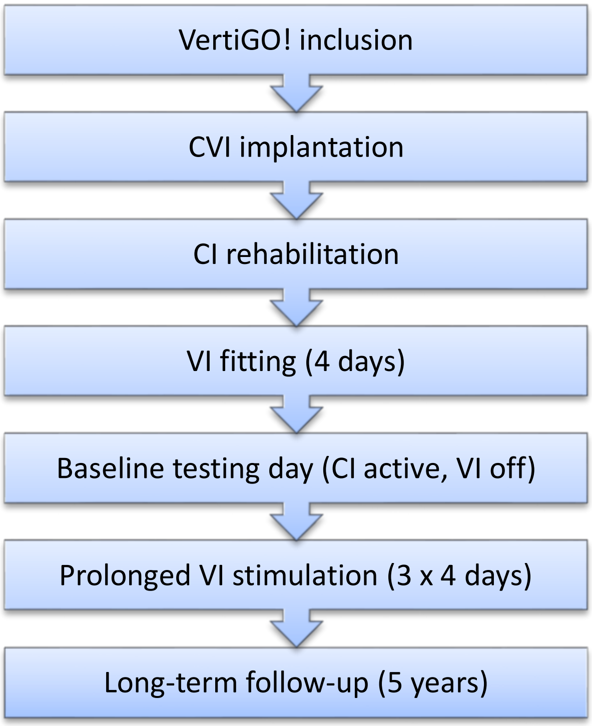

Figure 4. Overview of the VertiGO! trial

differentiating between the regular time investment for receiving a CI and the added time investment associated with the VertiGO! trial. The schedules are meant as an indication and may still be subject to slight changes which will be reported in the trial master file during the conduct of the study.

Within the detailed planning of the different testing periods, the time allocated for each test is deliberately given with a large margin. This gives ample time to thoroughly explain the tests and allows for frequent breaks while conducting the tests. Special care is taken to prevent overburdening the patients. This will provide a comfortable situation for the patient and achieve the most reproducible results.

The time frame in which the baseline testing and prolonged stimulation period will be conducted is deliberately chosen to be large (18 months). The goal is to implant all patients within the period of a year. This will allow for the fitting of both the CI and the VI of all the patients to be completed before any patient starts with the baseline testing and prolonged stimulation. The time period between the baseline testing and prolonged stimulation period for each individual patient will be kept short to ensure the validity of the baseline measurements.

Experience with previous CVI patients has shown that the fitting of the VI electrodes stays stable over several years of intermittent acute stimulation. Therefore we hypothesize that having a larger period of time between VI fitting and prolonged VI stimulation does not influence VI efficacy. To verify our hypothesis, the VI fitting will be checked the first day of every prolonged stimulation week.

By first fitting all the patients before the prolonged stimulation periods start, the information of the VI fitting can be used by the research team to establish an informed decision on whether the proposed trial set-up and trial activities can be conducted as envisioned. This will be based on what kind of vestibular and auditory responses can be elicited by the CVI. Therefore, any potential variation between patients in how the trial is conducted will be reduced, improving data quality. A more detailed description of this interim assessment is given in chapter 10.4.

Table 1. Overall schedule including an estimated time investment calculation (version 2)

OVERALL SCHEDULE (estimated time in min)			CI inclusion	VertiGO inclusion	OR Implantation	Post-op	CI rehabilitation											CI check	CI check	CI check	CI check	CI check	CI check
		Years					0											0,5	1	2	3	4	5
		Months	var.	var.	-1		0				1				2		3	6	12	24	36	48	60
		Weeks	var.	var.	-4	-3	0	1	2	3	4	5	6	7	8	10	12						
**VertiGO! testing periods** (see separate schedules for more details)																	VI FITTING 4 days	VI FITTING 4 days; BASELINE TESTING 1 day; PROLONGED STIMULATION PERIOD 3 weeks (4 days/week)	BASELINE TESTING 1 day; PROLONGED STIMULATION PERIOD 3 weeks (4 days/week)	BASELINE TESTING 1 day; PROLONGED STIMULATION PERIOD 3 weeks (4 days/week)			
Implantation		ENT consult	15			15																	
		Surgery			180																		
		Recovery			1 day																		
Device check		ART (VI+CI)			During OR						30							30*	30*	30*	30*	30*	30*
		Acute stimulation functional test							60					60			60						
		Impedance			During OR		0[A]	0[A]	0[A]	0[A]	0[A]			0[A]			0[A]	0[A]	0[A]	0[A]	0[A]	0[A]	0[A]
CI rehabilitation		Audiologist (fitting)	60				120	60	60	60				60			60[B]	30	30	30	30	30	30
		Speech therapy (rehab)	60					45	45	45	45	45	45	45	45	45	45						
		Social worker (consult)	60								60							60	60				
Imaging		CT and MRI	90																				
		CBCT				15													15	15			15
Auditory		Unaided hearing tests	30				30											30*	30*	30*	30*	30*	30*
		Aided hearing tests	30						30					30				30	30	30	30	30	30
		SPIN															15	15	15	15	15	15	15
Vestibular tests	Reflex	Caloric test		30																			
		o-VEMP		15													15*						
		c-VEMP		15													15*						
		Rotatory chair		15																			
		3D HIT		30																			
	Functional	DVA		60																			
	Follow-up	VBR																	60	60	60	60	60
		Test battery (to be determined)																120	120	120	120	120	120
Trial administration		Adverse Events			x	x	x	x	x	x	x	x	x	x	x	x	x	x	x	x	x	x	x
		Device deficiencies implant[C]			x	x	x	x	x	x	x	x	x	x	x	x	x	x	x	x	x	x	x
Subjective outcomes		Questionnaires		x		x											x	x	x	x	x	x	x
		Semi-structured interview															15						
Total time investment	Regular (CI)	Time (min)	345		2 days	15	150	105	135	105	105	45	135	45	45	45	105	150	150	90	90	90	90
		Visits	2		1	1	1	1	1	1	1	1	1	1	1	1	1	1	1	1	1	1	1
	Extra (VertiGO!)	Time (min)		165		15	30		60		30			60			105	165	240	240	225	225	240
		Visits		1		0											0	0	0	0	0	0	0

+ 17 days of testing periods between 3 and 24 months (±8h/day including breaks: see separate schedules)

Legend for overall schedule

Standard test for CI	XX (green)
Extra test for VertiGO! trial	XX (blue)
Standard visit for CI	(green cell)
Extra visit for VertiGO! trial	(blue cell)
Planning is variable	var.
If response was absent at preivous timepoint and no further optimized measuring techniques are available, repeated measurements will be skipped	x*
Part of CI fitting	A
Often clinically necessary	B
Device deficiencies of AMP processor will only be recorded during active use, namely during VI fitting period and period of prolonged VI stimulation	C

Table 3. Example of the schedule for the VI fitting period (version 2)

VI FITTING

Time	Day 0	Day 1	Day 2	Day 3	Day 4	Day 5
08:00		Breakfast	Breakfast	Breakfast	Breakfast	Breakfast
09:00		Costas array-based experiment	CI and CVI aided DVA		Static CI aided CNC + SPIN	
09:30			First fitting - Third SCC (1/2)	Second fitting - Second SCC (1/2)	Combined fitting evaluation and adjustment	
10:00		First fitting - First SCC (1/2)				
10:30			Break	Break	Break	
11:00		Break	First fitting - Third SCC (2/2)	Second fitting - Second SCC (2/2)	Combined fitting evaluation and adjustment	
11:30		First fitting - First SCC (2/2)				
12:00			Lunch	Lunch	Lunch	
12:30		Lunch				
13:00			Second fitting - first SCC (1/2)	Second fitting - Third SCC (1/2)	Combined fitting evaluation and adjustment	
13:30		First fitting - Second SCC (1/2)				
14:00			Break	Break	Break	
14:30		Break	Second fitting - first SCC (2/2)	Second fitting - Third SCC (2/2)	Costas array-based experiment	
15:00		First fitting - Second SCC (2/2)				
15:30			Semi-structured interview	Familiarization with gait and balance tests	Final overall fitting check	
16:00		VBR				
16:30					Static CVI aided CNC + SPIN	
17:00						
18:00	Dinner	Dinner	Dinner	Dinner	Dinner	
19:00						
Night	Hotel	Hotel	Hotel	Hotel	Hotel	

Table 2. Example of the schedule for the baseline testing day (version 1)

BASELINE TESTING

Time	Day 1
08:00	Questionnaires at home
09:00	Perception platform
10:00	Gait and balance tests (1/2)
11:00	**Break**
11:30	Gait and balance tests (2/2)
12:30	Rotatory chair
13:00	**Lunch**
14:00	DVA + static and dynamic aided CNC
15:00	**Break**
15:30	3D HIT + fHIT
17:00	
18:00	
19:00	
Night	**At home**

Legend for VI fitting and baseline testing schedules

		Colour
Time at the hotel		light blue
Time at the hospital	Testing	green
	Fitting	yellow
	Interview	dark green
	Free time	pale grey-green

Table 4. Example of the schedule for the prolonged stimulation period

PROLONGED STIMULATION PERIOD																				
Stimulation algorithm 1 (randomized)							Stimulation algorithm 2 (randomized)							Stimulation algorithm 3 (randomized)						
Sun.	Mon.	Tue.	Wed.	Thu.	Fri.	Sat.	Sun.	Mon.	Tue.	Wed.	Thu.	Fri.	Sat.	Sun.	Mon.	Tue.	Wed.	Thu.	Fri.	Sat.
1	2	3	4	5	6	7	8	9	10	11	12	13	14	15	16	17	18	19	20	21

Weekly schedule							
Day	Sunday 1	Monday 2	Tuesday 3	Wednesday 4	Thursday 5	Friday 6	Saturday 7
Time 08:00		Breakfast	Breakfast	Breakfast	Breakfast	Breakfast	
		Start-of-day check[C]	Start-of-day check[C]	Start-of-day check[C]	Start-of-day check[C]		
09:00		Habituation[A]	Habituation	Habituation	Habituation		
10:00			Perception platform	Gait and balance tests (1/2)	Perception platform		
		Break					
11:00		Head impulse tests[B]	Break	Break	Break		
			Interview	Gait and balance tests (2/2)	Rehabilitation		
12:00		Lunch	Lunch	Lunch	Lunch		
13:00		DVA	DVA	DVA	DVA + dynamic CNC		
14:00		Break	Break	Break	Interview		
		Rotatory chair					
15:00		Break	Rehabilitation	Rotatory chair	Break		
		Physiotherapeutic diagnosis		Costas array	Head impulse tests[B]		
16:00	Perception platform[E]		Break				
		Break		Static CNC			
17:00		End-of-day check[D]	End-of-day check[D]	End-of-day check[D]	End-of-day check[D]		
18:00							
	Dinner	Dinner	Dinner	Dinner	Dinner		
19:00							
Night	Hotel	Hotel	Hotel	Hotel	Hotel	At home	At home

Legend for prolonged stimulation period schedule		
Time at the hotel		
Time at the hospital	Testing	
	Rehabilitation	
	Interviews	
	Habituation and device check	
	Free time	
Including a short fitting check if necessary		A
3D HIT, fHIT, simulated 3D HIT and simulated rotatory chair		B
Impedance and UCL check		C
Impedance, UCL check and VAS-based list of questions		D
Measured without CVI active		E

4. STUDY POPULATION

4.1. Population (base)

The study population consists of capacitated adults who have (1) disabling symptoms of BV and (2) severe SNHL in at least the ear to be implanted. As part of standard clinical routine at the ENT department of the MUMC+, every patient that is believed to suffer from a balance disorder or is candidate for cochlear implantation undergoes a screening of his or her vestibular function by means of bilateral caloric test. Patients who appear to be eligible for and interested in participating in the next VI study are asked for consent to be put on a “VI-waiting list”. This list now includes hundreds of patients who are potentially fit for inclusion in this trial. The VI-waiting list will serve as the population base from which patients will be recruited.

In previous VI-related studies, patient inclusion has not been an issue. If it does turn out that including 8 patients out of the MUMC+ database proves problematic, potential patients might be sourced via collaboration with the Antwerp University Hospital (UZA). Patients from the UZA will be informed by their own ENT specialist who then refers them to a member of the research team for further information. All trial related activities will be conducted within the MUMC+.

4.2. Inclusion criteria

In order to be eligible to participate in this study, a subject must meet all of the following criteria:

4.2.1. *Vestibular*

Symptoms

- Chronic vestibular syndrome being presented by disabling symptoms of postural imbalance and/or impaired image stabilization (e.g. oscillopsia)

VOR function

- Reduced or absent bilateral VOR function based on at least one of the tests below meeting criteria A, with the other tests meeting criteria B:

Table 5. VOR function inclusion criteria

VOR function test	Criteria A	Criteria B
**Caloric response[1]**	Each side ≤6°/sec	Each side <10°/sec
**vHIT gain**	Bilateral horizontal SCC ≤ 0.6<br>1 Bilateral vertical SCC <0.7	2 Bilateral SCC <0.7
**Rotatory swing[2]**	Gain ≤ 0.1	Gain ≤ 0.2

[1] Sum of bithermal (30° and 44°) maximum peak slow-phase velocity
[2] Horizontal VOR function with sinusoidal stimulation on a rotatory chair at 0.1 Hz with Vmax = 50°/sec

BV etiology

- Onset of bilateral vestibular loss after the age of 2
- Vestibular dysfunction from a peripheral origin or idiopathic BV
- Patent vestibular end-organ (judged by CT)
- Vestibular function and symptoms have not recovered beyond inclusion criteria within 6 months from onset of symptoms including a 3 month rehabilitation program off vestibular suppressant medications

4.2.2. *Auditory*

- Meeting CI-candidacy in ear to implant with CVI
- Agreed to receive a MED-EL CVI implant with MED-EL sound processor

4.2.3. *General*

- Capacitated adults ≥ 18 years
- Proficient speaker of the Dutch language
- No contra-indications for CVI surgery
- Active participation in the trial related procedures such as regular testing, the VI fitting period, the baseline testing day and three weeks of intensive VI rehabilitation and testing in the study center (MUMC+) including an exercise regimen
- Agreed not to swim or to use or operate vehicles, heavy machinery, powered tools or other devices that could pose a threat to the participant, to others, or to property throughout the period of VI activation and until at least 1 day after VI deactivation

Remark: Patients who qualify to receive a regular CI as part of standard clinical care will have a preferential position to be included in the trial.

4.3. Exclusion criteria

A potential subject who meets any of the following criteria will be excluded from participation in this study:

4.3.1. *Vestibular/auditory*

- Signs of central vestibular/cochlear dysfunction or structural vestibular/cochlear nerve pathology (judged by physical examination / MRI)
- Clear signs of structural nerve pathology or indications of improperly functioning vestibular/cochlear nerves
- Requirement for electric-acoustic activation of the CI part (e.g. “hybrid” processor) prior to completion of the prolonged VI stimulation period
- Having received a cochlear implant earlier on the side to implant (e.g. explantation/reimplantation)
- Having received a cochlear implant from another brand than MED-EL in the other ear (e.g. bilateral implantation with different brands is not supported)
- Unwillingness to stop the use of antihistamines which might suppress VOR responses (e.g. cinnarizine) in the period of 1 month before until after each measurement point.

4.3.2. *General*

- Pre-lingual onset of bilateral profound deafness (< 4 years of age)
- Active participation in another prospective clinical trial
- Pregnancy or having plans to become pregnant at the time of imaging or during the VI trial
- Orthopedic, ocular, neurologic or other non-vestibular pathologic conditions of sufficient severity to confound vestibular function tests used in the study
- Current psychological or psychiatric disorders that could significantly interfere with the use or evaluation of VI stimulation
- Physical or non-physical contraindications for MRI or CT imaging prior to surgery
- Making chronic use of psychiatric medication which suppresses VOR responses (e.g. SSRI's, benzodiazepines)
- Significant dental problems which prohibit the stable use of a 'bite bar' (used as calibration reference for the gyroscope functionality of the CVI).
- Any medical condition, judged by the research team, that is likely to interfere with a study candidate's participation in the study

4.4. Sample size calculation

The main outcome parameter of the trial will be DVA (e.g. visual acuity while walking on a treadmill). Regarding the first primary objective (e.g. efficacy of prolonged VI stimulation), a comparison will be made between the condition with the CVI active and the condition with the VI-part inactive (whilst the CI remains active). Visual acuity (VA) can be expressed in several different units. However, for data analysis it is important to use the logarithm of the minimum angle of resolution (logMAR), since this unit scales linearly with the geometric progression of the visual acuity chart [42].

Data from previous measurements on the influence of the VI on DVA was used for the sample size calculation [25]. Although this data is obtained with a different version of the VI (which a.o. did not allow simultaneous VI and CI stimulation), it is the closest analog available to approximate the results of this trial. To account for the between-patient difference in VA, the data from the previous experiment, expressed in logMAR, were normalized to the static VA measured for each patient. This yields mean logMAR values of 0.065 (SD 0.061) and 0.205 (SD 0.066) for VI active and VI inactive, respectively. Since the size of the dataset is too small to establish whether or not the data is normally distributed, the sample size will be calculated for a two-sided Wilcoxon signed-rank test. Significance level (alpha) is set at 0.05 and power is set at 0.80 [43,44]. Both effect size and minimal sample size were calculated with G*Power 3.1.9.7 [45]. Effect size was determined to be 2.20, which resulted in a minimal sample size of 5.

Given the available resources (finances and available implants) and to give this statistical background a safety margin, this trial will aim to include a minimum of 8 patients, with a maximum of 13 patients. If a patient drops out of the trial after implantation, they will not be replaced due to the limited amount of implants that are available. By including at least 8 patients, we will have a safety margin for potential patient drop-outs which will ensure that we will still have enough data to prove a statistically significant change in DVA. By including more patients, we might gain more insight in the role of etiology on stimulation efficacy and interindividual differences in stimulation responses since this still small but

unique and significant population of VI recipients will pave the way towards clinical indications in the future

5. TREATMENT OF SUBJECTS

5.1. Investigational product/treatment

To provide prolonged simultaneous stimulation of both the vestibular organ and hearing organ a research device will be used: the CVI, which is presented in Figure 5. A more detailed description of the CVI can be found in section D of the standard research file (the IMDD). The basis of the CVI is a standard CI and its operating principles are the same: transferring information to the brain by means of electrical stimulation of sensory nerves. However, instead of only stimulating the auditory nerves in the cochlea, the CVI will also stimulate the nerves of the SCCs in the vestibular system. The CVI consists of two units: the implant and the external processor.

5.1.1. *The implant*

The basis of the CVI implant is a MED-EL CI implant. The implant consists of a coil for receiving radio frequency signals from the external processor, a magnet to allow for attachment of the external sending coil of the processor unit to the subcutaneous receiving coil of the implant and an array of 12 electrodes. In the normal CI implant these electrodes are arranged along one branch, designed to all be inserted into the cochlea. In the CVI implant, the three most basal electrodes are separated from the main electrode branch into three individual branches. These three individual branches are designed to be inserted into the SCCs to allow for stimulation of the vestibular system. The remaining 9 electrodes make up the cochlear array, designed to be inserted in the cochlea, analogous to a regular CI. Reallocating the

electrodes within the vestibulocochlear organ offers the opportunity to stimulate the vestibular nerves, while maintaining CI functionality in order to restore hearing.

Due to the similarities between the CVI and the cochlear implant, the CVI can be used in combination with a regular CI processor. In this case, the CI processor is programmed to only stimulate the 9 electrodes which are inserted into the cochlea, with the three vestibular electrodes turned off. This makes the CVI implant perform like a regular CI, without stimulation of the vestibular nerves. Outside of the VI testing periods, the patients who receive a CVI will be able to use the implant as a regular CI driven by a regular CI processor (described in chapter 7). During the VI testing periods, the patients will use the experimental CVI processor described in 5.1.2.

With the redesign of a standard CI implant into the CVI implant, the amount of intracochlear electrodes is reduced from 12 to 9, and the overall length of the cochlear array is reduced from 31 mm to 24 mm. It is difficult to make definitive statements on what influence these design changes might have on the CI performance of the CVI. Among the major CI manufacturers, there is no consensus on what the optimal electrode design is. Due to the differences in design philosophies between the companies, large differences in cochlear array designs exists. These designs include both straight and pre-curved arrays ranging in length from 18 to 31 mm containing between 12 and 22 electrodes [46]. These different designs yield large variations in the amount of stimulating electrodes, electrode spacing, angular insertion depth, cochlear coverage and frequency-to-place matching. However, a comparison study between the different brands yields only a 14% difference in overall performance [47], even though individually all these aspects have been shown to influence hearing outcomes.

Looking at the number of stimulating electrodes, research gives some broad outlines concerning its relationship with CI performance, although making comparisons is difficult due to large interpatient variability [48]. Based on theoretical knowledge of cochlear anatomy and signal transduction, increasing the amount of stimulating contacts in the cochlea should increase CI performance. However, this correlation does not appear to be so straight-forward. When looking at normal-hearing subjects, Shannon et al. [49] showed that speech recognition was mainly influenced by temporal information rather than spectral information. They demonstrated that, as long as enough temporal information was maintained in the signal, only a reduction of spectral information into 3 or less distinct spectral bands resulted in reduced speech discrimination. In other words: 4 electrodes distributed throughout the cochlea could theoretically be sufficient for near-normal speech discrimination. Looking at CI recipients, research into the number of active cochlear electrodes necessary for speech discrimination indicates that speech perception improves with an increasing number of electrodes, but plateaus around 8 electrodes [50,51], most likely due to current spread and increasing overlap in spectral stimulation. Along this line, Riss et al. [52] concluded that 4 electrodes are sufficient for simple listening tasks while more difficult tasks required up to about 8 electrodes, regardless of the used signal-processing strategy.

When reviewing the influence of cochlear array length on CI performance, conflicting results inhibit the drawing of definitive conclusions. Based on theoretical knowledge, a longer cochlear array should perform better due to the increase in frequency-to-place match and inter-electrode angular contact spacing [53]. This is substantiated by several studies, showing that utilizing the longest electrode possible which will fit

in a patient's cochlea results in the best performance [54,55]. However, contradictory studies found that by further inserting electrode arrays into the cochlea, and thereby increasing the frequency-to-place match and inter-electrode angular contact-spacing, no difference was observed in CI performance [56]. Deep insertion of a cochlear array has even been associated with reduced performance due to damage to the structures inside the cochlea [57,58]. When looking at the development of cochlear array designs over the past years, CI manufacturers seem to converge to a similar array length of between 20 and 25 mm, rather than making arrays which are as long as possible, compromising between cochlear coverage and causing structural damage [59].

The above-mentioned body of research is not capable of conclusively substantiating any claims regarding the influence of the redesigned CVI cochlear array on CI performance. Both the amount of electrodes and the array length are within industry standards and supported by literature. Preliminary results of research performed within our own consortium is indicating that there is no difference in performance between a regular CI and a CI set-up to mimic the CVI based on cochlear array length and amount of active electrodes (manuscript in preparation).

However, since a minimal negative effect of these design changes on individual CI performance cannot be ruled out a priori, patients will be informed about the design changes and their potential influences. Information on how cochlear array designs might influence performance is always given to the patient when receiving a standard CI, to help the patient in making an informed decision between the different CI manufacturers. Also the patient information brochure and informed consent form will include statements on the changed cochlear array design of the CVI compared to a standard CI.

5.1.2. *The processor*

To allow for stimulation of the vestibular nerves with information on head movement, the CI processor normally used with the CI implant is replaced by a research processor, the audio-motion processor (AMP). The AMP consists of two units: The audio processor, which is worn behind the ear, and the motion processor, which is worn on a cord around the neck. Both processor units are able to send information to the CVI implant using radio frequency signals via a set of coils. The internal coil is part of the implant, the external coil is connected via magnets to the internal coil and is worn on the skin behind the ear. The external coil housing also contains gyroscopes and accelerometers, which capture head motion in all directions. The external coil is connected by wire to the audio processor, which is in turn connected by wire to the motion processor. The motion processor also houses the battery.

Through the use of this combined device, both the balance and hearing organs can be stimulated simultaneously. Head motion captured by the gyroscopes and accelerometers is transferred to the motion processor where it is translated into electrical pulses. These electrical pulses are then transferred to the electrodes implanted inside each of the SCCs. This allows for the stimulation of the SCC afferent nerve branches, transferring the motion information to the brain. Using this input, the patient is able to again perceive head motion. Hearing is restored in the same way as is done with a regular CI. Sound is captured by a microphone housed in the audio processor unit, where it gets translated into electrical signals which are used to stimulate the auditory nerves inside the cochlea.

5.2. Use of co-intervention (if applicable)

In case of discomfort the CVI processor can be turned off or removed from the patient's head at any point, instantly stopping the stimulation of the vestibular system.

5.3. Escape medication (if applicable)

- Not applicable -

6. INVESTIGATIONAL PRODUCT

6.1. Name and description of investigational product

See chapter 5.1 for a general description of the investigational product and see section D of the standard research file (the IMDD) for a more detailed description.

6.2. Summary of findings from non-clinical studies

Extensive animal testing has been conducted to gain general knowledge on vestibular stimulation and to test vestibular implants [13]. This research identified electrical stimulation as an effective means of stimulating the vestibular system in animals already in the 1960's [60,61]. Currently, the main focus of animal research is directed towards thoroughly investigating the effects of electrical stimulation parameters on vestibular responses [62], focusing on vestibular-ocular responses [63–72], as well as orientation percepts and postural responses [73–75].

No non-clinical studies were performed with the CVI research devices. All the results were obtained from clinical studies, conducted over the last 13 years. Therefore there is no summary of findings from non-clinical studies specific to this investigational product.

6.3. Summary of findings from clinical studies

Clinical research on CVI's within the Geneva-Maastricht group started in 2007 with implanting a CI device with a single channel VI [13]. After development of additional surgical techniques [14–16], and successful intra-operative vestibular stimulation [17], the first CVI's containing multichannel VI's were implanted in 2012. The possibility of acute hearing preservation and the feasibility of additional surgical tools were presented [18,19]. It was shown that the CVI is a safe and effective way of stimulating the vestibular system, even in a heterogeneous patient population [13,20–24]. Relevant functional benefits were shown by restoring the VOR, improving vision during walking [25] and by restoring high frequency DVA [76].

Recently the preliminary findings of a prolonged vestibular stimulation trial conducted in the USA by the group of Della Santina were published [32]. In this trial a VI-only device is being tested on patients without severe SNHL. They have reported follow-up results on their first four patients after several years of continuous daily stimulation. Their results show stable restoration of the VOR and positive subjective outcomes. Alongside this they have not reported any adverse effects of the prolonged stimulation. With the VertiGO! trial we aim to fill research gaps which will not be answered within the Della Santina trial, conducting a randomized and more rigorous trial focused on the first weeks of prolonged stimulation, including CI functionality during vestibular stimulation and general device safety focusing on a different patient population (BV and severe SNHL).

6.4. Summary of known and potential risks and benefits

6.4.1. *Risks related to the CVI implant*

MED-EL (manufacturer of CI systems) has established and maintains a documented and effective Quality Management System in accordance with ISO 13485 and applicable regulatory requirements. MED-EL's Quality Management System (QMS) outlines the processes necessary to fulfil national and international

requirements for medical devices. The CVI is a device developed for research and is therefore not a CE marked medical device, nor is it intended to be put on the market.

Production of the CVI research implants takes place in environmentally-controlled cleanrooms for products that are distributed in sterile condition. Electrostatic-sensitive assemblies and subassemblies are constructed in ESD-protected areas. Qualified and calibrated equipment is used in production and for testing/verification purposes. MED-EL implements procedures to ensure that if the work environment can adversely affect product quality, it is adequately monitored and controlled. Personnel involved in the manufacturing of research devices are adequately trained for research device production and generally trained according to MED-EL's quality management system.

Patients will be informed, that with this implant (just as with many other implantable devices in medicine), the patient should not undergo MRI scanning. The implant has not been tested for MRI safety and is labeled as MRI Unsafe (see Annex IV of the IMDD, section D of the standard research file). If there are medical reasons for the patient to undergo MRI examination, the patient is required to first contact the ENT department of the MUMC+ to discuss the risks and the possibilities.

A more detailed description of the device related risks can be found in section D of the standard research file (the IMDD).

6.4.2. *Risks related to the AMP*

The AMP is a research use only device designed to allow simultaneous stimulation of the SCCs and the cochlea. It will be used under supervision of medical personnel while the patient is free to move around and perform routine daily activities. The audio processor part of the AMP is equivalent to a standard CI processor. The motion processor is added onto this, following the same design principles and stimulation restrictions as a CI processor to ensure safe stimulation of the CVI implant. To perform the fitting for the motion-processing part of the AMP, the AmpFIT software package is used, specifically designed to work together with the AMP.

This will be the first time the AMP will be used to drive a CVI implant in humans. To minimize any potential risks, the AMP will only be used by the patient when the patient is under supervision inside the hospital. Also, the patient is able to stop the stimulation of the CVI implant by the AMP at any point by turning it off or taking off the device. This immediately stops any current from passing through the CVI implant.

A more detailed description of the device related risks can be found in section D of the standard research file (the IMDD).

6.4.3. *Risks related to CVI surgery*

Until now, none of the 13 patients that were implanted with a CVI by the Geneva-Maastricht group suffered from any surgical complications [13] related to the well-defined surgical approach [16]. Risks of surgical implantation are low. Surgery is derived from a frequently performed surgical intervention which has proven to be safe: cochlear implantation [77]. The main risk of CVI surgery is damage to the sensory structures in the cochlea and the vestibular organ, resulting in hearing loss and loss of vestibular function. These risks are minimalized by only implanting patients with severe SNHL in the ear to be implanted and

with only minimal vestibular function remaining. Alongside this, the potential hearing loss is compensated for by the CI functionality of the CVI.

The inclusion criteria imply that three types of patients are eligible for participation: patients with BV ánd bilateral deafness who have an indication for reimbursement of unilateral cochlear implantation (in the Netherlands), patients with BV ánd unilateral deafness for whom cochlear implantation is not insured (in the Netherlands) and patients with BV ánd bilateral deafness who have already received a CI in the contralateral ear. Since the CVI is a combined device, combining a VI- and a CI-part in one device (Figure 5), hearing will be restored on the implanted side. Therefore in the first group (bilateral deafness), the CVI will be implanted instead of a regular CI implant during their cochlear implantation in the same surgical field, without additional incisions. The two other groups (unilateral deafness and bilateral deafness with already one CI implanted) will undergo surgery specific for this trial, but will also benefit from the CVI implantation: their hearing will most likely improve [78].

6.4.4. *Risks of explanting the CVI*

Until now, none of the previous 13 patients implanted with a CVI, needed to be explanted. However, since the CVI is a derivative of the CI, the reasons for explanting (e.g. infection) and the associated risks should be equal to that of a CI. It is shown that although CIs are not often explanted, it is a safe procedure with low risks [79]. The only risks are related to the explant surgery itself: infection and bleeding. Since the CVI is only implanted in organs which have a complete or near total loss of function, there will be no substantial risk of damaging these organs. Another reason for explanting might be due to device failure, followed by implanting a new device (which could be either a CVI or a CI). This will not be performed for patients as part of this trial, and it should be noted that explanting and implanting a new device is not a goal. The concept of a CI, and by analogy also a CVI, is that it can provide lifelong stimulation.

6.4.5. *Risks of electrical stimulation of nerves*

From research and years of experience with cochlear implantation it is known that long-term stimulation of nerves does not lead to unwanted neural damage, as long as current is kept below a safe level and the right materials are used [80]. In our project, a modified CI is used, which implies a well-tested, daily used device with standard electrodes. It is called “modified” since it has four arrays of electrodes instead of one. The use of multiple cochlear electrode arrays (e.g. “split array”) is a known clinical procedure that can be applied in certain cases where significant cochlear calcification requires more than one cochlear insertion site to reach the cochlear nerve. Previous trials of the Geneva-Maastricht group already demonstrated that acute responses to VI stimulation can be obtained after an extended time (at least several years), without damaging the nerves [13].

6.4.6. *Risks related to adjusting the settings of the CVI*

Adjusting the settings of the CVI results in changing the previous stimulation state of the vestibular nerves. Research shows that this can lead to dizziness [81]. By performing the adjustments of the stimulation settings in a gradual fashion, utilizing the adaptive capabilities of the human brain, this dizziness can be kept to a minimum. Additionally, due to the change in balance perception, there is a potential risk of falling. The current set-up of the trial takes these risks into account and attempts to minimalize them by

scheduling 4 full days to adjust and fine-tune the stimulation settings. This fitting period specifically focusses on preventing side effects as much as possible. When applied in previous research from the Geneva-Maastricht Group, this way of adjusting the stimulation settings resulted in only mild and temporary side effects (e.g. tiredness)[17].

It has also been shown that side effects decrease with increasing use of the CVI. Previous experiments with a patient who received a CVI showed that the duration of unwanted nystagmus which can cause dizziness decreased substantially within several on-off cycles [17]. This suggest the potential of reaching a situation of dual-state adaptation, where the patient is able to switch the CVI on and off without any side effects [63,80].

Since this will be the first time that the CVI will be used for prolonged stimulation while the patient performs daily tasks, there is a chance for side effects such as nausea to occur during this period, in combination with certain stimulation algorithms. Also there is a potential risk of falling due to changes in balance perception. When they occur, these side effects might be reduced by optimizing stimulation settings. These side effects can always be stopped by turning off the AMP or taking off the device. The risks related to these side effects are minimized due to the vestibular stimulation only being applied under supervision within the hospital.

6.4.7. *Benefits of the CVI*

Patients who receive a CVI will in any case benefit from the cochlear part of the device: their hearing will most likely be improved. If vestibular stimulation via a CVI works as is hypothesized and such a device would be available for long term use, it can lead to a substantial quality of life improvement for patients. Disabling symptoms associated with BV would be reduced and a patient's ability to participate in society would be improved. These benefits will however not currently be available for patients, since the AMP is a research-only device, and will only be used for research purposes under supervision in the hospital. This research can lead to further development of the concept of a VI, potentially leading to a device which will be available for patients to use as part of standard clinical care.

6.5. Description and justification of route of administration and dosage

- Not applicable -

6.6. Dosages, dosage modifications and method of administration

The CVI implant is implanted inside the cochlea and the SCCs, with the receiver coil being placed subcutaneously. The AMP is connected to the implant via a sending coil placed on the skin on top of the receiver coil and is held in place by magnetic force. The audio processor part of the AMP is placed behind the ear and the motion processor part of the AMP hangs on a cord around the patient's neck.

6.7. Preparation and labelling of Investigational Medicinal Product

A full description of device production and examples of labelling can be found in section D of the standard research file (the IMDD).

6.8. Drug accountability

The CVI implants will be delivered by courier to the hospital, in analogy with the standard delivery of CI implants. The AMP devices will be delivered by the manufacturer to the research personnel, and will stay inside the hospital until the end of the trial, at which point they will be returned to the manufacturer.

7. NON-INVESTIGATIONAL PRODUCT

7.1. Name and description of non-investigational product(s)

Outside of the VI fitting period and the prolonged stimulation period when the patient is under supervision in the hospital, the patient will use a regular CI processor to drive their CVI implant. This processor is the CE-marked MED-EL Sonnet 2 audio processor. Being the latest version of the MED-EL CI processor product line, it utilizes advanced hearing technology to give optimal sound quality. Supplementary documents describing the Sonnet 2 audio processor can be found in section D6 of the standard research file.

Utilizing the standard MED-EL MAESTRO software package the three electrodes placed in the SCCs will be turned off. The deactivation of electrodes is part of the normal CI fitting procedure, performed when for example faulty electrodes are detected or when electrodes are not situated in the cochlea due to the electrode array not being fully inserted into the cochlea. By deactivating the vestibular electrodes, the CVI implant operates exactly like a regular CI implant with its 3 most basal electrodes turned off.

7.2. Summary of findings from non-clinical studies

- Not applicable -

7.3. Summary of findings from clinical studies

- Not applicable -

7.4. Summary of known and potential risks and benefits

- Not applicable -

7.5. Description and justification of route of administration and dosage

- Not applicable -

7.6. Dosages, dosage modifications and method of administration

The Sonnet 2 audio processor is worn behind the ear with the sending coil being placed on the skin on top of the receiving coil of the CVI implant, held in place by magnetic force.

7.7. Preparation and labelling of Non Investigational Medicinal Product

Labelling will be as standard for this CE marked device.

7.8. Drug accountability

- Not applicable -

8. METHODS

8.1. Study parameters/endpoints

The study parameters are divided into main study parameters and secondary study parameters. Main study parameters include all objective outcomes included in the trial. Secondary study parameters include all subjective outcomes. An overview of the outcome parameters and endpoints in relation to the objectives is given in table 6.

8.1.1. *Main study parameters/endpoints*

The primary objective is assessing the efficacy of the CVI in restoring vestibular function. This will be measured by using a set of vestibular tests, of which DVA will be the main outcome parameter. The tests that will be performed observe two vestibular-related reflex pathways: VOR (gaze stabilization) and VSR (postural balance). The VOR tests consist of the DVA test, the functional head impulse test (fHIT), the 3D video head impulse test (3D HIT) and the torsion swing test. All these tests quantify the VOR at different frequencies and at different levels of functionality. The VSR is observed based on gait and balance tests, looking at the full body response to vestibular stimulation on a functional level. Besides these tests, also the threshold of the perception of movement will be measured using the perception platform.

The main comparison will be made between the measurements done during baseline testing (VI-OFF) and the measurements done during the prolonged stimulation period (VI-ON) (see chapter 2). This will give a

Table 6: Overview of VertiGO! objectives, goals, endpoints and outcome parameters

<table>
<tr><th colspan="3" rowspan="2">Objective</th><th rowspan="2">Goal</th><th rowspan="2">Measurement points</th><th colspan="3">Outcome parameters</th></tr>
<tr><th colspan="2">Type</th><th>Test</th></tr>
<tr><td rowspan="8">Primary</td><td rowspan="8">Prolonged daily stim.</td><td rowspan="4">1a</td><td rowspan="2">Overall efficacy</td><td rowspan="2">VI-OFF (baseline) vs
VI-ON (stim. weeks)</td><td rowspan="3">Prim.</td><td>Main</td><td>DVA</td></tr>
<tr><td rowspan="2">Aux.</td><td rowspan="2">fHIT, 3D HIT, Torsion Swing,
Gait analysis, Perception platform</td></tr>
<tr><td rowspan="2">Adaption to stimulation</td><td rowspan="2">VI-ON (start stim. weeks) vs
VI-ON (end stim. weeks)</td></tr>
<tr><td>Sec.</td><td></td><td>Questionnaires, Interviews</td></tr>
<tr><td>1b</td><td>Safety</td><td>stim. weeks</td><td colspan="3">(S)AE's</td></tr>
<tr><td rowspan="3">1c</td><td rowspan="3">Efficacy of
3 stim. types</td><td rowspan="3">VI-ON (week 1) vs
VI-ON (week 2) vs
VI-ON (week 3)</td><td rowspan="2">Prim.</td><td>Main</td><td>DVA</td></tr>
<tr><td>Aux.</td><td>fHIT, 3D HIT, Torsion Swing,
Gait analysis, Perception platform</td></tr>
<tr><td colspan="2">Sec.</td><td>Questionnaires, Interviews</td></tr>
<tr><td rowspan="6">Secondary</td><td rowspan="2">Rehab.</td><td rowspan="2">2a</td><td rowspan="2">Rehab. program</td><td rowspan="2">stim. weeks</td><td colspan="2">Prim.</td><td>Gait analysis</td></tr>
<tr><td colspan="2">Sec.</td><td>Questionnaires, Interviews</td></tr>
<tr><td rowspan="3">CI</td><td rowspan="3">2b</td><td rowspan="2">Interaction between CI and VI</td><td rowspan="2">CI-ON/VI-OFF (baseline) vs
CI-ON/VI-ON (stim. weeks)</td><td colspan="2">Prim.</td><td>Aided CNC, SPIN, vestibular test</td></tr>
<tr><td colspan="2" rowspan="2">Sec.</td><td rowspan="2">Questionnaires, Interviews</td></tr>
<tr><td>CI as part of VCI</td><td>CI-ON (up to 5 years)</td></tr>
<tr><td>Long term</td><td>2c</td><td>Acute efficacy</td><td>VI-ON (up to 5 years)</td><td colspan="2">Prim.</td><td>Subset of vestibular tests</td></tr>
</table>

general result on the efficacy of prolonged vestibular stimulation. A further statement of CVI efficacy will be made based on the response to stimulation throughout the different prolonged stimulation weeks. Therefore the VOR tests will be performed several times during the prolonged stimulation weeks. A comparison of these results will yield statements on potential adaptation effects (e.g. habituation to receiving information via the CVI, increase/decrease in response gain and potential increase in VOR alignment). The same set of VOR and VSR tests measured throughout the prolonged stimulation weeks will also serve as parameters for comparing the different stimulation algorithms.

A subset of the vestibular tests will be performed during the annual follow-up visits. These parameters will be used to quantify the stability of acute stimulation and implant functionality over a clinical follow-up period of 5 years.

The functionality of the CI-part of the CVI will be assessed with an aided consonant-nucleus-consonant (CNC) hearing test. This will be done by making the comparison between aided CNC scores using the regular CI processor and aided CNC scores using the CVI processor (the AMP) with both CI and VI part active. During the CI follow-up visits speech in noise (SPIN) tests will be performed as part of the auditory follow-up test set to allow for evaluation of the CI part of the CVI implant and compare this outcome with CI performance of a regular CI implant.

8.1.2. *Secondary study parameters/endpoints*

The patients' subjective experience with the CVI will be quantified using both questionnaires and a number of semi-structured interviews at different points during the study duration. An overview of the time points at which questionnaires and interviews will be administered can be found in table 1, 2, 3 and 4 (chapter 3) and chapter 8.3. The results of these questionnaires and interviews will allow us to more extensively describe our patient population, quantify the impact that both the CI and the VI functionality have on patient well-being, characterize the perceived benefit the CVI will pose to the patient and allow us to see if the CVI meets the patients' expectations. All this data will also be vital for creating a health technology assessment (HTA), which will aid the further development of the concept of vestibular stimulation.

Previous research within our group[29] (partly as-of-yet unpublished) has shown the diversity of reported symptoms among BV patients. These previous interview studies have shown the importance of characterizing all the different subjective aspects and processes on which the CVI can have an effect. Alongside this, based on clinical experience during the previous VI trial in Maastricht, the patients which are eligible for inclusion into the trial have a personal drive to share their story and give their opinion. Therefore, we wish to channel this drive into quantifiable results by using an elaborate set of subjective outcome measures. The questionnaires and interviews will be spread out over the full trial duration, and across several days during the stimulation weeks, to reduce the burden on the patient

All questionnaires are described in chapter 8.3.5 and can be found in section F1 of the standard research file.

8.2. Randomization, blinding and treatment allocation

During the implantation, CI fitting, VI fitting and baseline testing the researchers and patients will not be blinded since these procedures will be identical for all patients. However, during the prolonged stimulation period patients will be blinded to which type of stimulation algorithm is used. The three types that will be tested will each be randomly assigned to one of three 4-day prolonged stimulation periods. This way the effect of different stimulation algorithms can be assessed both within patients and between patients.

The sequence of stimulation algorithms will be randomized and single-blinded. Randomization will be performed by using a block randomization-based design [82]. Since there are 3 stimulation algorithms which will be randomized over 3 time periods, each algorithm appearing only once, there are 6 distinct algorithm sequences. To equally distribute all the patients over 6 algorithm sequences a block size of 6 is used. A custom “urn” will be created in the following way: Each of the 6 possible algorithm sequences is added to the urn once. Additional algorithm sequences are chosen at random without replacement from the total of 6 algorithm sequences and also added to the urn. This is done until the amount of algorithm sequences in the urn is equal to the amount of patients which will be included. The urn now contains as many allocation possibilities as there are patients, with each allocation possibility featuring at least once and no more than twice. Randomization will be performed in the period after VI fitting and before the prolonged stimulation period. When an allocation is assigned to a patient during randomization, this allocation is removed from the urn. Therefore, the urn will be empty after all patients have been allocated. This way all possible algorithm sequences will be allocated to at least one patient without offering predictability of algorithm sequence allocation at any moment.

8.3. Study procedures

8.3.1. *Description of the vestibular tests which will be conducted*

Caloric test

The caloric test uses temperature to create fluid flow inside the SCCs which results in deformation of the cupola and subsequent increased or reduced firing of the SCC nerves. This results in an eye movement response which is captured with ENG. A change in temperature is achieved by irrigating the ear canal with water of a specific temperature. It is one of the most used tests clinically to assess VOR response [83]. Eye movement calibration is performed before each irrigation. Patients are positioned in a supine position with their head tilted 30° from the horizontal plane. Each irrigation lasts 30 seconds with a volume of at least 250 mL water for cold (30°C) and warm (44°C) irrigations. A 5 minute stimulus interval is kept between irrigations.

Dynamic visual acuity test (DVA)

The DVA test is described in the article by Guinand et al. [25]. It is aimed at measuring a functional aspect of VOR: visual image stabilization while walking. The DVA test consists of the patient walking on a treadmill, walking at different walking speeds, with or without holding the handrails. While walking, they will perform a visual acuity test by reading out Sloan optotypes (CDHKNORSVZ) displayed in decreasing size on a computer screen in front of the patient. Using an adaptive up-down staircase procedure, the LogMAR

of each patient is determined. LogMAR (logarithm of Minimal Angle of Resolution) is used as an estimate of visual acuity. Patients are also tested in static condition (while standing still) to serve as a reference. The test takes around 10 minutes, depending on the patient.

Since the patients are walking under controlled conditions on a treadmill, this moment is also taken to assess gait. Two MOX physical activity monitor accelerometers are attached to the patient’s upper leg and lower back so the movement the lower limb and center of mass can be observed. The data obtained from these MOX sensors serve as complementary to the full gait analysis performed at a different moment. The addition of these accelerometers do not influence the patients gait [84] and therefore will not influence the outcome of the DVA.

When DVA is measured during the baseline testing day, a maximum safe walking speed will be established. This speed will also be used during the prolonged stimulation period to be able to compare visual acuity. During the DVA tests conducted within the prolonged stimulation period one additional measurement is added to the DVA test. While the patient is using the treadmill for the DVA measurement, the patient will also again be asked to walk at their maximum safe walking speed. The change in walking speed will also be taken as an outcome parameter to indicate an increase in dynamic balance.

3D Head impulse test (3D HIT)

The HIT is one of the standard tests performed daily as a diagnostic tool for patients with vestibulopathy in our clinic. The test is designed to quantify VOR at its fundamental level by measuring eye moment (gain and direction) as a response to passive high velocity head movements. It is performed along three different rotational axes: horizontal, right-anterior-left-posterior (RALP) and left-anterior-right-posterior (LARP). The patient is seated and fitted with VOG goggles to measure eye movements. The patient is asked to keep their eyes fixated on a target 1.5 meters ahead of them and to blink as little as possible. The examiner stands behind the patient and while holding the head of the patient, moves the head several times fast (>150°/s for horizontal, >100°/s for RALP and LARP) with a small amplitude (±20°) randomly in either direction of one rotational axis. This is repeated for each rotational axis. Frequent brakes are taken between the head impulses to allow the patient to blink. Depending on the patient, this test takes around 20 minutes to complete.

Functional head impulse test (fHIT)

The fHIT is a variation of the 3D HIT, aimed at measuring the VOR at the same frequency as the 3D HIT, but at a more functional level. It quantifies how well a person can observe an image during high velocity passive head movements. The patient is seated and a gyroscope is fitted to the patients head by means of a head band. A screen is placed in front of the patient at a distance of 1.5 meters and the patient is asked to look at a fixation target shown on the screen. Before the start of the fHIT, the static visual acuity threshold is acquired by the fHIT system. After that, The examiner stands behind the patient and moves the patients head in the horizontal plane, in the same fashion as is done during the 3D HIT test (velocity >150 °/s, amplitude = ±20°). The gyroscope which is attached to the patient’s head registers the head movement and a Landolt optotype ring is displayed for 80 ms on the screen, 80 ms after head movement velocity exceeds 10 °/s. After the head movement the patient is asked to choose the orientation of the

Landolt ring from a set of 8 orientations by pressing the corresponding direction on a keyboard. The test is repeated 32 times (16 times per side) and takes around 10 minutes.

Torsion Swing test

The torsion swing test is aimed at quantifying the VOR response at a fundamental level, similar to the 3D HIT. However, with the torsion swing test the head movement stimulus has a smaller acceleration and a lower velocity compared to the 3D HIT. Therefore it quantifies the VOR response at a lower movement frequency. It is part of the standard tests performed daily as a diagnostic tool for patients with vestibular hypofunction in our clinic. The torsion swing test is performed using horizontal sinusoidal rotation (0.1Hz) with a peak velocity of 60°/s, while the subject sits in a rotating chair in complete darkness. Eye movements are recorded with VOG goggles fitted to the patients head. The test takes around 10 minutes to complete

cVEMP and oVEMP

Cervical vestibular evoked myogenic potentials (cVEMPs) and ocular vestibular evoked myogenic potentials (oVEMPs) are measurements of the VSR and VOR responses, respectively. They are routinely used in clinics and research to diagnose vestibular disorders due to their ability to selectively observe these reflex pathways at a fundamental level. The VEMP measurements are used to observe any residual vestibular function during the trial (if present).

cVEMPs are measured over the sternocleidomastoid muscle after stimulating the ipsilateral vestibular organ with multiple transducers: inserted earphones, bone conduction, galvanic stimulation (only at screening). Subjects are in a supine position with their back tilted in an angle of 30° from the horizontal plane and are asked to turn their head away from the location of the stimulus and to lift their head up slightly. A visual feedback system connected with a monitor ensures correct muscle contraction, measured with electromyography (EMG) software and self-adhesive electrodes. Two hundred EMG traces with a minimum mean rectified voltage (MRV) of 60 µV and a maximum MRV of 205 µV are accepted.

oVEMPs are measured over the inferior oblique muscle after stimulating the contralateral vestibular organ with the same stimulation parameters as for cVEMPs. Subjects are in a supine position and are instructed to keep their eyes fixed on a focus point 30 degrees behind the head to achieve superomedial gaze. A minimum of 300 EMG traces are accepted.

Perception platform

A hydraulic platform is used to measure the perceptual self-motion thresholds within each subject. A twelve-option paradigm, six translations and six rotations, is delivered by the platform. The subject sits in complete darkness in a chair mounted on the platform, fastened by a seatbelt, while wearing a blindfold and headset to mask visual and auditory cues. The six translations include motions in the horizontal plane (forward, backward, right, left) and in the vertical plane (up and down). The rotations includes yaw left, yaw right, pitch forward, pitch backward, roll left and roll right. Thresholds are found using a staircase protocol with a maximum duration of one hour. Motion trials starts at the highest possible acceleration and their directions are randomly chosen by the examiner. After each motion, the subject is asked to inform the examiner about the type and direction of the perceived movement. In case the subject can

indicate the correct type and direction of the movement, the stimulus is decreased with $0.1 m/s^2$ (translations) or $10°/s^2$ (rotations). In case of an incorrect response, the acceleration is increased with 0.05 m/s^2 or $5°/s^2$. If the subject then could indicate the correct type and direction of movement, the acceleration is decreased with 0.03 m/s^2 or $3°/s^2$. A double confirmation of the lowest threshold combined with double incorrect responses at the acceleration one step below threshold, is considered the perceptual self-motion threshold for that motion profile.

Gait and balance tests

To observe the VSR response at a functional level, patients will complete a number of walking trials on the computer assisted rehabilitation environment (CAREN) system to assess their walking patterns, stability and biomechanics during various tasks. It allows for the quantification of biomechanical measures such as stepping behavior, gait stability and gait variability.

The CAREN is a dual belt treadmill system with a 6 degree of freedom motion platform and a 180 degree virtual reality environment. Reflective markers will be placed on anatomical landmarks and will be recorded via the system's 3D motion capture system. Additionally, two MOX accelerometers will be placed on the lower back and thigh to record lower limb and center of mass accelerations during the tasks.

At the beginning of the session, the system and set-up will be explained to the patients, as well as the measurement protocol. The session will start with familiarization trials, during which the patients will walk on the treadmill for short periods at multiple speeds to become accustomed to walking on the system. During all sessions, the subjects will be secured with a safety harness connected to an overhead frame. The CAREN operator will ensure that the subject's knees cannot reach the ground and that the subject cannot walk too far to the front or back of the treadmill. The exact amount and characteristics of the familiarization period is flexible due to the heterogeneous nature of the patient group and their various experiences with treadmill walking. Once the CAREN operator, patient and clinical researcher are in agreement that the patient is comfortable and familiarized to the set-up, the measurement will be conducted. These measurements include walking at different speeds in combination with different perturbations of the walking platform. Standardized questions will be included regarding effort, fatigue and difficulty for each task to allow comparison between what the patient perceives during the measurements and their gait performance.

Beside the walking trials on the CAREN system, patients will also complete a set of clinical balance and gait tests (e.g. the mini-balance evaluation systems test (mini-BESTest)) [85,86]. Clinical balance and gait tests are often used as a diagnosis tool or to assess progression during physiotherapeutic treatment in clinics. They allow for the functional characterization of both gait and balance during walking and standing, offering insights into the VSR at a more functional level than the walking trials on the CAREN system. By including both types of tests, a more complete characterization of any potential change in gait and balance caused by vestibular stimulation can be performed.

Costas array-based experiment

To assess the fundamental response of the brain to SCC nerve stimulation, a Costas array-based experiment will be conducted. The results of this experiment will give valuable insights in how the brain

processes electrical stimulation and will allow for the development of more optimized stimulation algorithms. The experiment consists of stimulating the SCCs with a signal based on a Costas array. This will induce eye movements, which will be recorded using VOG while the patient sits in a completely dark room. The exact response of the eye movements is correlated with the input signal, allowing for the exact evaluation of the eye movement response to a signal with stimulation intervals that are fully uncorrelated to other parts of the original signal.

Evoked Vestibular Brainstem Response

The electrically evoked vestibular brainstem response (eVBR) is the response of the vestibular nerve as a result of electrical stimulation, all the way up to the brainstem, equivalent to the well-known auditory brainstem response (ABR)[117]. The vestibular implant will be used to evoke the response, making it possible to quantify the response, and potentially relate it to VI output. This not only gives a fundamental insight in the processing of vestibular information by the vestibular nerve and related brainstem areas, but proving this concept might open doors for applications as intra-operative electrode positioning verifications and using it as basis for CVI fitting, where it is contrary to the VOR and VEMP not impaired by anesthesia or patient cooperation.

An eABR equivalent setup will be used, consisting of a system controlling the CVI stimulation (the clinically used MAESTRO software controlling the MAX-box (both by MED-EL, CE-approved)) and a system designed for measuring ABR (the clinically used Surpass (EMS medical, CE-approved)). Responses will be measured on the three vestibular electrodes (eVBR) and a selection of the cochlear electrodes (eABR). The subject is asked to lay fully relaxed, and does not need to do anything during the test. Intersubject variability and the reproducibility of the traces will be used as main outcome measure for the feasibility of the method. Stimulus related effects are determined by a step-wise change in stimulus characteristics, e.g. stimulus amplitude.

8.3.2. *Description of the hearing tests which will be conducted*

Consonant nucleus consonant (CNC) test

Speech understanding in quiet will be assessed using the Dutch CNC test [87]. This clinical test is part of the selection, rehabilitation and follow-up of CI patients in the Netherlands. Phoneme scoring (%correct) in a quiet free field listening situation at a distance of 1m to the loudspeaker will be evaluated. Two conditions will be measured: static and dynamic. Static refers to the standard clinical setting whereby the subject is sitting in a chair in front of a loudspeaker (no vestibular stimulation). Dynamic refers to a setting specifically designed for evaluation of the combined CVI function whereby the subject is walking on a treadmill (active vestibular stimulation) (see also DVA for the description of a comparable set-up).

Speech in noise (SPIN) test

In this test sentence materials such as the Leuven Intelligibility Sentence Test (LIST) [88], the Flemish[115]/Dutch version of the Matrix test [89] or number materials such as the Digits-in-noise test (DIN)[116] are presented in noise. An adaptive procedure is applied whereby the noise is fixed at a level of 65dB sound pressure level (SPL) while the speech level is varied. This results in the speech-reception-threshold (SRT), the signal-to-noise ratio at which the subject is able to still understand 50% of the sentences

correctly. Speech and noise will be presented in free field from the same loudspeaker in front (S0N0). This test can only be performed meaningfully if a minimum level of speech understanding in quiet is achieved (e.g. 40-50%).

8.3.3. *Description of the device tests which will be conducted*

Auditory nerve response telemetry (ART) measurements

ART measurements are a way to measure electrically evoked compound action potentials (ECAPs) of both vestibular and cochlear nerves with the CVI implant. ECAPs can be used to characterize nerve responses to electrical stimulation, which will offer insights into current spread throughout the inner ear and which may, among other things, be used as a guide to optimize the fitting of the CVI [18]. To perform ART measurements, the CVI implant will be connected to a computer which runs CI fitting software. The measurement does not require any actions to be performed by the patient and takes around 5 minutes.

Impedance

Impedance measurements act as a general safety check of the device. The test is performed to detect problems with the structural integrity of the electrodes and their ability to conduct electricity. This test is a routine test for CI implants and works the same for CVI implants. It is performed by connecting the implant to a computer and measuring the impedance of each electrode. The patient does not experience anything from this measurement, which takes around 2 minutes including set-up.

8.3.4. *Description of the imaging techniques which will be used*

Magnetic resonance imaging (MRI)

To assess anatomy and patency of the inner ear including the vestibular organ MRI imaging is used. The results of the MRI scan are used to decide whether the patient is fit for receiving a CVI. MRI is also a part of the regular pre-operative tests for receiving a CI.

Computed tomography (CT) scan

In combination with the MRI scan, also a CT scan of the mastoid is clinically included in the pre-operative assessment for receiving a CI . During the pre-operative assessment for CVI implantation, we will use the same pre-operative CT to assess the individual anatomy and patency of the inner ear, and to plan a surgical approach for the CVI surgery. The pre-operative mastoid CT will also be fused with the per-operative 3DCT to assess electrode placement during the surgery. The radiation dose of a standard mastoid CT-scan is around 0.6mSv. See chapter 13.1.11 for further information on the total radiation exposure, the associated risks and justification for the risks.

Cone beam computed tomography (CBCT) scan

A CBCT scan allows to check the position of the CVI electrodes in the cochlea and SCCs. In other clinics, this is often already a standard procedure after cochlear implantation. In our clinic, a post-operative CBCT scan is often included when clinical research is performed involving CI's ("ELEPHANT Trial" ABR 51559, "Bimodal Zoom" ABR 64874). Also within clinical care it is generally applied when problems or complaints occur with regular CI devices. The CBCT scan involves only a very small dose of radiation (0.05 mSv). See

chapter 13.1.11 for further information on the total radiation exposure, the associated risks and justification for the risks.

8.3.5. *Description of the questionnaires and interviews*

All questionnaires can be found in section F1 of the standard research file.

Patient population

A set of standardized, validated questionnaires will be used to describe the characteristics of the patients included in the trial. This set includes the falls efficacy scale international (FES-I) [95], the dizziness handicap inventory (DHI) [96], the oscillopsia severity questionnaire (OSQ) [97,98] and the hospital anxiety and depression scale (HADS) [102]. These questionnaires quantify the severity of the different symptoms associated with BV and a utility based QoL assessment.

Furthermore we will also incorporate a BV specific questionnaire which is still under development within our group (aimed to be completed around the start of 2021). This questionnaire is based on interviews with [30] and focus groups of BV patients. It aims to characterize all aspects of how BV affects a patient's life and well-being (manuscript in preparation). Although the scope and formulation of the questions included in this questionnaire are expected to stay the same, the selection of questions still has to be finalized. The resulting questionnaire is expected to be about half of the one currently submitted in section F1 of the standard research file.

HTA

To allow for the calculation of quality adjusted life years (QALYs) related to receiving a CVI, a set of questionnaires are included at several time points throughout the trial. These questionnaires include the euroqol five-dimensional questionnaire (EQ5D-5L) [100], the icepop capability measure for adults questionnaire (ICECAP-A) [101] and the health utility index mark 3 (HUI-3) [10,99]. . These questionnaires will be administered before implantation, after CI rehabilitation and during the VI stimulation period to assess both the influence of the CI and the VI part of the CVI device. The QALY calculations will be valuable for the creation of a HTA, exploring the feasibility of a CVI device from a societal and economic standpoint.

Hearing

Complementary to the objective hearing related outcomes, also a patient reported hearing questionnaire will be administered during the different CI rehabilitation and follow-up visits: the speech spatial and qualities of hearing scale (SSQ-12) [103,104]. By using both objective and subjective outcome measures to quantify CI functionality, a more complete characterization can be achieved.

Tinnitus

To monitor any potential change in tinnitus due to CVI surgery the tinnitus questionnaire (TQ) [105] will be administered before and after surgery. A change in tinnitus severity (both positive and negative) is a common side-effect of CI surgery [106,107], which indicates the importance of quantifying tinnitus also during this trial.

Daily VAS-based list of questions

At this moment a specific measurement tool for quantifying the impact of a CVI on subjective outcome parameters has not yet been developed. To reduce the amount of questionnaires the patient has to fill in, and to be able to better quantify day-to-day differences in subjective outcomes during the prolonged stimulation period, a shorter, more specific, custom-made list of VAS based questions was created by our research team. This VAS-based list of questions aims to quantify the influence of the CVI on the self-perceived severity of BV and hearing loss related symptoms, alongside characterizing patient satisfaction with the CVI.

Interviews

At the end of the CI rehabilitation period, during the VI fitting period and the prolonged stimulation weeks semi-structured interviews will be conducted with the patients. The goal of these interviews is to collect patient experiences on the use of the CVI and obtain expectations about the potential benefit of the CVI in daily life. Although less structured and quantifiable than questionnaires, these interviews will allow us to characterize more subtle and personal changes. Furthermore, the patient perspective will be valuable for substantiating the claim of further developing the concept of VI stimulation.

Rehabilitation

To give more structure to the rehabilitation program which is included in the prolonged stimulation period, the patient-specific functional scale (PSFS) [108] questionnaire will be used to set and monitor patient-specific training goals. The goals will be characterized both before using the VI-functionality of the device and during the prolonged stimulation period.

8.3.6. *Description of the trial visits*

A detailed overview of the trial planning can be found in chapter 3, table 1.

Inclusion

At the ENT department of the MUMC+, every patient that is believed to suffer from a balance disorder or is candidate for cochlear implantation, undergoes a screening of his or her vestibular function by means of bilateral caloric test as part of the standard clinical routine. This has resulted in a large cohort of patients on a “VI-waiting list” that could be eligible for and have indicated to be interested in taking part in the next VI study, and patients are still being added to this list. All these patients gave consent to be contacted by one of the research team members, which was written down in the clinical patient file.

When ethical approval is obtained, one of the members of the research team will contact the patients on this “VI-waiting list” either via email or post. The patient will be asked whether they want to receive more information regarding this specific study. If so, the patient information brochure will be sent to the patient via post. If a patient who is in the clinic for a regular visit appears to be eligible for participation in the trial, the patient will be informed about the trial by their doctor or audiologist. If the patient is interested in learning more about the trial, the patient information brochure will be handed to the patient in person. Via the patient information the patient will be informed that he or she can contact an independent physician or the members of the research team if the patient has any questions. The patient will be asked to reply either by email or telephone. If the patient is interested in participating, a meeting will be

scheduled with a member of the research team (who is not the primary physician) to discuss the patient information, to allow the patient to ask questions and to sign the informed consent form. Every candidate will have at least 2 weeks to think about taking part in the study after receiving the patient information brochure.

After obtaining the candidate's written informed consent, a complementary extensive vestibular examination will be done. This VI-inclusion visit will consist of a list of standard vestibular examination tests which are daily, routinely used to investigate the vestibular function in patients with balance disorders. The tests which are included in this list are the caloric test, the oVEMP, the cVEMP, a 3D HIT and a torsion swing test. One extra test, specifically related to the CVI study, will be added: the DVA test. A description of these tests is given in chapter 8.3.1. The complete VI-inclusion visit includes around 3 hours of testing, unless patients were already extensively tested as part of their diagnostic work-up for their pathology. If so, all previously performed tests will be skipped and the VI-inclusion visit will take less time.

Alongside this, a standard CI inclusion procedure will be conducted to assess the patient's eligibility for receiving a CI in the ear to be implanted. This includes hearing tests, an ENT consult and visits to an audiologist, a speech therapist and a social worker. Also an MRI and a neuronavigation CT scan will be made. Any CI eligibility testing that might have been done before as part of regular clinical care will not be repeated.

If the patient meets all inclusion criteria and none of the exclusion criteria as described in chapter 4.2 and 4.3, the patient will be included in the trial. A total of 8 patients will be included. A date will be assigned for the surgical procedure and the patient will be given a set of questionnaires to be filled in at home.

The surgical procedure (performed under general anesthesia):

1. Routine retro-auricular approach
2. Routine mastoidectomy and posterior tympanotomy (as performed in cochlear implant surgery)
3. Human fascia is harvested from the temporal muscle
4. The bed for the receiver is drilled (as performed in cochlear implant surgery)
5. The SSCs are blue-lined using the ampullar "V"-approach
6. The round window is drilled and opened to facilitate cochlear implantation
7. The cochlear electrode is implanted using the round window approach
8. Electrodes are implanted in each canal:
    a. The canal is fenestrated near the ampulla
    b. The electrodes are inserted in the canal to reach the ampulla
    c. Fluoroscopy is used to optimize the electrode positioning
    d. 3D-CT scanning is performed and images are fused with the preoperative CT scan and MRI to verify the electrode positioning. If necessary, electrodes are repositioned using fluoroscopy. If necessary, a second 3D-CT scan is performed to check the electrode positioning after repositioning.
    e. The electrodes are fixed using 3M™ Ketac™
    f. A final 3D-CT scan is performed to assess electrode positioning after fixation.

   g. The fenestration is closed with bone pate and the harvested fascia
9. The receiver is fixated (as performed in CI surgery)
10. The wound is closed in three layers (as performed in CI surgery)
11. Structural integrity is checked as performed in CI surgery by performing ART and impedance measurements. Stimulation potency is checked by performing a function test of the vestibular electrodes: during stimulation, VOR is recorded using videogoggles.
12. A standard head dressing is applied
13. The procedure is completed and the patient wakes up
14. One night is spent in hospital and the next day the head dressing is removed and the patient is discharged from hospital (as in CI surgery)
15. One week post-operatively, the stitches are taken out, the healing cap is removed and the wound is checked at the outpatient department (as in CI surgery)

Post-operative period

Around 1 week after surgery (combined with the ENT post-surgery check-up) the patient will undergo a CBCT scan in order to evaluate the location of the electrodes in the ampullae and cochlea. The patient will also be asked to fill in the TQ questionnaire.

CI rehabilitation

CI rehabilitation will be performed as standard in our clinic. This includes a minimum of 11 visits (can be individually extended if necessary) in the time-span of 3 months to establish a fitting and to train the patient with using their CI. The CI rehabilitation period starts around 1 month after implantation. During the first visit a first fitting will be established and the patient will receive their standard CI processor (described in chapter 7), which will be connected to the CVI implant.

Each of the CI rehabilitation visits will take between 1 and 2 hours. During each CI visit, the impedances of all VCI electrodes will be checked. On top of that a short functional test of the VI electrodes will be introduced. This functional VI test will first be piloted in one subject. If the procedure is accepted, the intention is to repeat this functional VI test for each of the upcoming subjects as an extended part (max 60 min.) to three of the regular CI visits. This functional VI test will entail a short, maximum 2 seconds, stimulation of the VI electrode in each of the SSC, starting low in amplitude and increasing in small steps. To generate this stimulation the CI fitting software Maestro will be used. After each stimulation the patient will be asked if they felt something, what they felt and if it is comfortable. As soon as the patient experiences the stimulation as uncomfortable, the test will be stopped. Aside from the subjective feedback, also the eye movements will be captured objectively using video goggles. At no time during this function VI test, or any other moment during CI rehabilitation, the AMP processor will be turned on. The only time patients will actively use the AMP processor is during the VI fitting period and the prolonged stimulation period while they are being supervised in the hospital by the research team.

The final CI fitting visit, 3 months after the start of the CI rehabilitation period will include extra trial related procedures in addition to regular CI procedures. At this point ART measurement will be performed. Also cervical and ocular VEMPs are measured, if they were not absent during inclusion. Alongside this a SPIN hearing test is performed and the patient is asked to fill in questionnaires.

VI fitting

An example of the VI fitting schedule can be found in chapter 3, table 2. Just as with a regular CI, a fitting will have to be established for the VI part of the CVI. During the fitting the correct minimum and maximum current levels at which to stimulate the vestibular nerves will be established. This will only be done once the intensive CI rehabilitation is completed and a stable CI fitting is established (i.e. circa 4 months after implantation). During this trial, for the first time in the context of VI research by the Geneva-Maastricht group, three VI electrodes will be combined with simultaneous CI stimulation. Therefore we have allocated 4 days to establish a fitting of the full CVI, allowing for an accurate fitting process including an ample amount of breaks to keep the burden on the patient as low as possible. Especially when the VI electrodes are stimulated for the first time, the patients perceive it as a strange experience which they have to get used to. During the fitting procedure patients will be staying in a hotel next to the hospital, to reduce the burden of traveling. Also breakfast, lunch and dinner will be paid for. If the patient prefers to distribute the fitting days into a larger period (for example 2 days of fitting per week for 2 weeks), this will also be possible. Although we believe 4 days is more than sufficient to create a CVI fitting, due to its novelty, in specific cases it might be necessary to add another day of fitting. This will be reported upon in the trial master file as a protocol deviation.

The goal of the fitting is to establish for each of the vestibular electrodes the threshold (T) of stimulation which yields a vestibular response and the upper limit of stimulation which is comfortable (UCL) for the patient. The T level will be based on perception, VOR or VSR, whichever occurs at the lowest stimulation current. UCL is defined as the lowest current level at which either stimulation becomes no longer comfortable for the patient based on a VAS scoring system, the stimulation elicits unwanted responses (e.g. tickling, stimulation of the facial nerve or pain) or the maximum safe current that can be supplied by the implant is reached. The range between these two points is called the dynamic range (DR), and establishing a DR for each electrode is the same as what is done during a regular CI fitting. The fitting of each VI electrode will be done in two stages to fine-tune the fitting, as shown in chapter 3, table 2. Due to current spread, the amount of current reaching certain nerves might be higher with combined stimulation than with individual stimulation. Therefore, the last step in the fitting process is to evaluate the UCL of all electrodes during combined stimulation, adjusting stimulation levels if necessary. Also a hearing test will be conducted to evaluate CI performance while both the vestibular and cochlear electrodes are active.

During the fitting period an interview will be conducted with the patients to characterize in a structured way the patient’s expectations of the CVI and how they describe their first experiences with vestibular stimulation. Also 1,5 hours will be reserved for a visit to the balance and gait lab in the hospital, to get acquainted with some of the tests that will be performed during baseline testing and the prolonged stimulation period. This will help ensure more accurate test results later on, besides allowing the patient to be introduced to the physiotherapists and motion scientists that will be working with them.

The fitting period will also be used to characterize the first responses of the vestibular system to electrical stimulation. These responses will be elicited by low level stimulation using a Costas array-based

stimulation sequence and recorded for stimulation optimization. It will be measured both at the start of the fitting period and at the end, to observe early adaptation processes to vestibular stimulation.

Baseline testing

In the period of 6 to 24 months after the start of the CI rehabilitation the baseline testing and the prolonged stimulation period will be conducted. The full schedule of the baseline testing day can be found in chapter 3, table 3. The CI and VI fitting will be completed for all patients before the first patient starts with the baseline testing and prolonged stimulation (see chapter 3.2 and 10.4). The maximum time period between baseline testing and prolonged stimulation will be kept short (in the order of a few months) to ensure that the results from baseline testing serve as a good control for the prolonged stimulation period.

During the baseline testing day, the control measurements (VI-OFF) will be conducted for all vestibular outcome parameters. This includes the DVA, 3D HIT, fHIT, torsion swing, perception platform and gait and balance tests. The 3D HIT, fHIT and torsion swing tests are performed twice in a row: Once with an active CI and once without. This way the potential influence of CI stimulation on vestibular function and vice versa can also be characterized. Alongside the vestibular tests, an aided CNC hearing test with the regular CI processor will also be conducted.

The patient will also be asked to fill in a set of questionnaires at home and bring them to the hospital during the baseline testing day. At the end of the day the patient will be asked to fill in the VAS-based list of questions.

Prolonged stimulation period

The prolonged stimulation period consists of 4 days of stimulation per week, for 3 weeks. A full schedule of the prolonged stimulation period is given in chapter 3, table 4. Each stimulation period is scheduled from Monday to Thursday. During this time, the patient will be staying in a hotel next to the hospital (i.e. from Sunday afternoon until Friday morning) and spend their days from approximately 08:30 until 17:30 in the hospital. Friday until Sunday are reserved as a resting period at home.

During the prolonged stimulation period the patients will only use the CVI processor while they are under supervision by a member of the research team in the hospital. This means that patients will make use of the CVI around 8 hours per day, for 4 days in a row. At any other time they will use their own CI processor, only providing CI functionality without vestibular stimulation. The schedule of testing for each week is kept the same. The main parameter that will change between the different weeks is which stimulation algorithm will be used (see figure 6): (A) baseline stimulation with motion modulation (B) reduced baseline stimulation with motion modulation (C) baseline stimulation with no motion modulation. Baseline stimulation consists of constant stimulation of the vestibular nerves at 50% of DR. Reduced baseline stimulation consists of constant stimulation of the vestibular nerves lower than 50% of DR. Motion modulation consists of stimulation of the vestibular nerves based on angular head movement. The parameters (e.g. level of baseline stimulation and modulation) of each paradigm will be determined after analyzing the results of VI fitting. The sequence of stimulation algorithms will be single blind and randomized for each patient. In this way, the patients will serve as their own control.

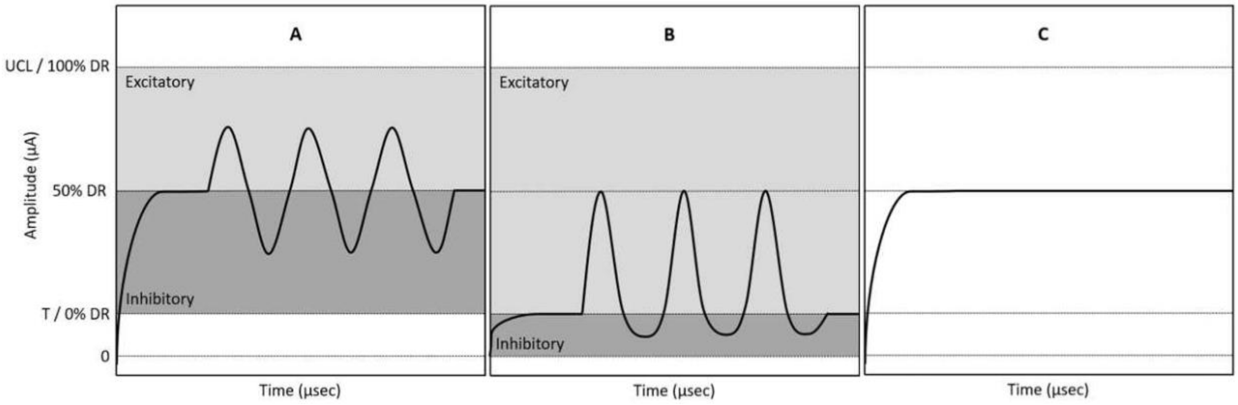

Figure 6: Stimulation algorithms used during the trial. (A) baseline stimulation with motion modulation (B) reduced/no baseline stimulation with motion modulation (C) baseline stimulation with no motion modulation.

For all activities during the prolonged stimulation week, ample time is reserved to keep the feasibility of the schedule high and the burden on the patients low. For the same reason a considerable amount of breaks are included in the schedule. During this time the patient can rest and perform leisure activities like reading or walking around within the hospital.

Each day of CVI stimulation will start and end with impedance measurements and a check of UCLs. This is done as a safety precaution to make sure that the implant is performing as expected, even though it is expected that these parameters will not change during the prolonged stimulation period. These measurements will take only 5 minutes to complete. After this, one hour is reserved on Monday and half an hour on every following day to allow the patient to get used to the stimulation. Previous research by the Geneva-Maastricht group has shown that habituation to stimulation at 50% of the DR takes around 30 minutes for the first time the nerves are stimulated, and progressively less time at the start of each consecutive stimulation period [13]. By taking this time every morning for the patient to get used to the stimulation, the reproducibility of the measurements will increase. At the start of every day the patients will also be asked if any (S)AE's occurred after leaving the hospital the previous day (i.e. attacks of nausea or vertigo). At the end of each prolonged stimulation day, the patients will be asked to fill in the VAS based list of questions to characterize their experiences with the CVI on a daily basis.

All the tests which will be conducted during the prolonged stimulation weeks are shown in table 4 of chapter 3 and described in chapter 8.3.1 through 8.3.5. All testing will occur during the four days of prolonged stimulation, except for three perception tests, one performed each Sunday afternoon before every period of prolonged stimulation. The patient will be asked to arrive in the hospital around 16:00 on Sunday for 1 hour of testing. The patient will be tested while using their regular CI processor. The goal of the tests is to assess if any change occurs in the baseline measurement after the patient has performed the perception platform test a few times. This is to exclude any chance that a learning effect might occur for the perception platform test, which is something that has not yet been fully studied. At the end of this test the patient will also be asked to fill in the VAS based preference scales.

Besides testing, the patients will also follow a rehabilitation program for 3 hours every week. During the allocated time-slot every Monday an individual diagnosis for each patient is established and the patient will be asked about their personal goals they would like to achieve in combination with the CVI using the PSFS questionnaire. Based on this a rehabilitation program will be set up and repeated every week in the same fashion. The main focus of the rehabilitation will be placed on achieving the personal goals of each patient and to challenge the patient in using their CVI to the fullest.

At the end of each prolonged stimulation period the patients are asked to fill in a set of questionnaires to gather specific information for the HTA and to evaluate CVI functionality for each stimulation algorithm.

CI follow-up visits

For the regular clinical CI rehabilitation care the patient is asked to visit the hospital at regular intervals for a check-up (see chapter 3, table 1). During these visits of around 2 hours another 4 hours are added on average to also perform a VI check-up. This VI check-up will include tests similar to the tests which were performed during the fitting week and/or prolonged stimulation period. During these tests the VI electrodes will be stimulated and response to stimulation will be characterized. The main purpose is to test whether electrode impedances and vestibular responses remain constant over time. Also during these visits additional hearing tests and questionnaires will be administered.

At the 1, 2 and 5 year follow-up visits a CBCT scan will be made to assess if any change occurs on the position of the electrodes inside the SCCs and the cochlea. This information can then be linked to device efficacy and it can be used to assess potential causes of problems with the CVI if they occur. It will also allow for evaluating the benefit from postoperative scanning in the follow-up of the CVI.

8.4. Withdrawal of individual subjects

Patients can leave the study at any time for any reason if they wish to do so without any consequences. The investigator can decide to withdraw a subject from the study for urgent medical reasons.

As stated in chapter 4.2 and 4.3, patients who make use of VOR suppressing medications will either be asked to stop using this medication before joining the trial (in the case of cinnarizine), or will not be included (in the case of psychiatric medication, e.g. benzodiazepines). However, it can occur that the patient will start taking these medications after they have been included in the trial. If this occurs, it will be noted as a confounder due to the potential effect these medications can have on the VOR response. It will not be seen as a reason for the withdrawal of the patient since the use of these medications does not affect the safety of the patient in the trial and excluding a patient is more detrimental to the usability of the overall data than the potential effect VOR suppressing medications might have. The patients will be notified that they have to inform the research team if they start using VOR suppressing medications (see section E of the standard research file).

Pregnancy is also stated as an exclusion criteria for the trial. However, pregnancy will also not be seen as a reason for the individual withdrawal of a patient. If a patient becomes pregnant, all trial activities which might pose a risk to the pregnancy will be postponed or cancelled until after the pregnancy. Postponing or cancelling will have a smaller influence on the usability of the data gathered in this trial than the

outright withdrawal of a patient. By individually changing the trial schedule there will be no risks for the patient or the pregnancy. The patients will be notified that they have to inform the research team in case of pregnancy (see section E of the standard research file).

Due to the novelty of this study, situations might occur which can cause unforeseen discomfort or side effects for the patient. If this occurs, for example caused by certain stimulation algorithms or measurements, the patient will always have the choice to not undergo a certain part of the trial activities. The planning will then be adapted to this patient specifically to decrease the burden on the patient, and this will not necessarily be seen as a reason for individual patient withdrawal.

A specific patient will be withdrawn from the regular trial activities if, after multiple fitting attempts, it is established that none of the three vestibular electrodes are able to elicit a vestibular response. This patient will however be offered a detailed examination of potential factors which might have caused this result (e.g. etiology, inner ear and nerve anatomy, electrode placement) and a follow-up plan will be made depending on these results. Unrelated to this follow-up plan, the patient will still receive all the regular care associated with receiving a CI.

8.4.1. *Specific criteria for withdrawal (if applicable)*

- Not applicable -

8.5. Replacement of individual subjects after withdrawal

Due to the limited number of implantable devices available, patients will not be replaced if withdrawal occurs after implantation. However, the possibility of a drop-out has been accounted for in the sample size calculation.

8.6. Follow-up of subjects withdrawn from treatment

Subjects who withdraw from the trial after implantation will still receive regular CI care, including the rehabilitation and check-up visits which are part of standard clinical practice. During these visits, the state of the implant is monitored as is normal with a regular CI implant. Any (S)AE's which might have occurred will be logged.

8.7. Premature termination of the study

The study will be halted or terminated prematurely if any of the below-mentioned situations occur:

- if the judgement of the competent medical research ethics committee that has assessed the study is irrevocably revoked;
- if a reasonable case can be made for terminating the study in the interests of the health of the research subjects;
- if it transpires that continuation of the study cannot serve any scientific purpose, and this is confirmed by the medical research ethics committee that has issued a positive decision on the study;

- if one of the parties or the funder has been declared insolvent or a bankruptcy/winding-up petition has been filed in respect of one of the parties or the financier, or one of the parties or the financier is dissolved as a legal entity;
- if the principal investigator is no longer capable of performing the tasks of the principal investigator, and no replacement agreeable to both parties can be found;
- if one of the two parties fails to comply with the obligations arising from the agreement and, provided compliance is not permanently impossible, this compliance has not taken place within thirty days of the defaulting party receiving a written request to comply, unless failure to comply is not in reasonable proportion to the premature termination of the study;
- if circumstances beyond the control of the sponsor, investigator or funder make it unreasonable to require the study's continuation.

9. SAFETY REPORTING

9.1. Temporary halt for reasons of subject safety

In accordance to section 10, subsection 4, of the WMO, the sponsor will suspend the study if there are sufficient grounds to suggest that continuation of the study will jeopardize subject health or safety. The sponsor will notify the accredited METC without undue delay of a temporary halt including the reason for such an action. The study will be suspended pending a further positive decision by the accredited METC. The investigator will take care that all subjects are kept informed.

9.2. AEs, SAEs and SUSARs

9.2.1. *Adverse events (AEs)*

Adverse events are defined as any undesirable experience occurring to a subject during the study, whether or not considered related to the experimental intervention or the trial procedure. All adverse events reported spontaneously by the subject or observed by the investigator or his staff will be recorded. Because primary trial objectives involve a safety assessment, the patients will also be actively asked during all trial visits if any AEs occurred.

9.2.2. *Serious adverse events (SAEs)*

A serious adverse event is any untoward medical occurrence or effect that

- results in death;
- is life threatening (at the time of the event);
- requires hospitalization or prolongation of existing inpatients' hospitalization;
- results in persistent or significant disability or incapacity;
- is a congenital anomaly or birth defect; or
- any other important medical event that did not result in any of the outcomes listed above due to medical or surgical intervention but could have been based upon appropriate judgement by the investigator.
- An elective hospital admission will not be considered as a serious adverse event.

The sponsor will report the SAEs through the web portal *ToetsingOnline* to the accredited METC that approved the protocol. When SAE's have occurred which indicate an inevitable life-threatening risk or which can lead to serious injuries or illness and which require immediate reparative action, the SAE will be reported within 2 workdays and no later than 4 calendar days after the sponsor has first knowledge of the SAE. This includes SAE's which lead to the (temporary) halt of patient inclusion, the (temporary) halt of the study or require changes to be made to the medical device. All other SAE's will be reported within 7 days after the sponsor has first knowledge of the SAE.

9.2.3. *Suspected unexpected serious adverse reactions (SUSARs)*

Since this study does not involve medicinal products, this section is considered to be non-applicable in the case of the current study.

9.3. Annual safety report

Since this study does not involve medicinal products, this section is considered to be non-applicable in the case of the current study.

9.4. Follow-up of adverse events

All AEs will be followed until they have abated, or until a stable situation has been reached. Depending on the event, follow up may require additional tests or medical procedures as indicated, and/or referral to the general physician or a medical specialist.

(S)AEs need to be reported till the end of study within the Netherlands, as defined in the protocol.

9.5. Data Safety Monitoring Board (DSMB)

As requested by the MERC azM/UM, a DSMB will be established to perform an independent interim analysis on preliminary data. The point at which this interim analysis will take place is described in chapter 10.4 of the research protocol (C1). The exact content, tasks and members of the DSMB is described in the DSMB charter (section K5 of the standard research file).

The advice(s) of the DSMB will only be sent to the sponsor of the study. Should the sponsor decide not to fully implement the advice of the DSMB, the sponsor will send the advice to the reviewing METC, including a note to substantiate why (part of) the advice of the DSMB will not be followed.

10. STATISTICAL ANALYSIS

The definitive statistical plan will be constructed after data has been collected for the first patient. Below follows the general outline of the statistical plan.

A single-subject repeated measures design will be used whereby each subject acts as their own control. The outcomes of the different parameters (as described in chapter 8.1) will be bootstrapped[109] and descriptively represented by stating mean and standard deviation, median/interquartile range and minimum/maximum values. Due to the small sample size, normality can most likely not be established, therefore non-parametric statistical tests will be performed. Confidence interval will be set at 95%. Due to the small sample size, the outcomes of this trial are not meant to serve as proof for population-wide claims. The goal of the trial is to assess feasibility of chronic vestibular stimulation with a focus on individual effectiveness (compared to within-subject control) and safety, rather than giving definitive statements on its effectiveness as a treatment for a group of patients.

Since the nature of the trial is exploratory and not confirmatory, the choice is made to include a full set of vestibular tests to assess efficacy of the CVI. The data from this trial will be used to be more rigorous in choosing outcome parameters for a future confirmatory trial with a larger population, in order to reduce multiplicity during that trial. Therefore, during this trial no structural statistical steps are taken to control the impact of multiplicities on the type 1 error. However, care will be taken to avoid combining the results of the different outcome measures into one statement on efficacy. DVA will serve as the main primary outcome for VI efficacy due to it giving objective results on the functional restoration of the VOR response pathway. All other parameters will be viewed as auxiliary.

If missing data are observed, the cause of missingness will be ascertained. When the cause of missingness is suspected to be structural, these cases will be described and excluded from analysis of the affected outcome measure. If data is concluded to be missing at random, the imputation strategy will be decided based on the nature and extend of the missingness. In this process the potential for reduced variance caused by interpolation-based imputation techniques will be taken into account. A final imputation strategy will be decided as part of the definitive statistical plan.

10.1. Primary study parameter(s)

The main comparison of the primary outcome parameters will be between the conditions of VI-OFF and VI-ON. The comparison between these two conditions will be done via an intention-to-treat approach using a Wilcoxon signed-rank test (non-parametric) due to the small sample size and the unlikelihood of being able to proof normality. For this analysis, data from the baseline testing, the prolonged stimulation period and the long-term follow-up will be used. To compare between the different stimulation algorithms, only descriptive statistics will be applied. The lack of statistical power caused by the small sample size does not allow for a multilevel repeated measures analysis of the data.

10.2. Secondary study parameter(s)

The secondary study parameters (i.e. questionnaires) will be analyzed in the same fashion as the primary study parameters, comparing the VI-OFF condition measured at baseline to the VI-ON condition at the

end of the prolonged stimulation period. Qualitative analyses, i.e. thematic/ content analysis, will be performed on the transcripts derived from the semi-structured interviews.

10.3. Other study parameters

- Not applicable -

10.4. Interim analysis

This trial will be the first time that our group will research prolonged vestibular stimulation and the first time both the CI and VI capabilities will be used simultaneously. Due to the novelty of this trial, an interim analysis will be conducted after five of thepatients have had their CI and VI fitted. As mentioned in chapter 3.2, the trial planning is designed to allow for this. Due to unknown factors caused by for example etiology, differences in anatomy or electrode placement, it is possible that not all of the vestibular electrodes will elicit a vestibular response. This might influence the usability of certain vestibular stimulation algorithms. The information on the fitting and early performance of the devices will give the research team clear insights in the feasibility of the proposed prolonged stimulation period, purely based on what response can be obtained from each of the vestibular electrodes. The decision on whether or not the trial can be continued with or without revisions will be based on the professional experience of the researchers involved in the trial. For example: if the fitting method needs to be revised, additional day(s) of VI fitting might be needed prior to entering the prolonged stimulation period.

The option for conducting an additional interim analysis is reserved if the results of the prolonged stimulation periods of the first patients indicate fundamental issues with, for example, one of the stimulation algorithms which warrant potential revisions.

A DSMB will join in the above-mentioned interim analysis, as mentioned in chapter 9.5. The exact content, tasks and members of the DSMB is described in the DSMB charter (section K5 of the standard research file).

For the above-mentioned interim analyses, no data will be used which will have to be unblinded. Therefore there is no need to set up a DSMB, based on the Guideline on Data Monitoring Committees published by the EMEA in 2005.

11. ETHICAL CONSIDERATIONS

11.1. Regulation statement
The study will be conducted according to the principles of the Declaration of Helsinki (2013 Version) and the Medical Research Involving Human Subjects Act (WMO).

11.2. Recruitment and consent
A detailed description of the inclusion procedure is given in chapter 8.3.6. During patient recruitment and while obtaining patient consent care will be taken to inform the patient as best as possible on the activities and risks involved in the trial. Also, the patients will be given ample time to make an informed decision. There will be an independent doctor which the participants are able to contact, if they have any questions. Her name and telephone number are given in the patient information brochure. This will allow the patient to make a well-informed decision which will be recorded clearly in an informed consent document, all according to the WMO regulations.

11.3. Objection by minors or incapacitated subjects
Minors and incapacitated patients will not be included in the study.

11.4. Benefits and risks assessment, group relatedness

11.4.1. *Individual patient benefits*
Patients who receive a CVI will benefit from the cochlear part of the device: their hearing will most likely be improved. Patients with bilateral SNHL who do not yet have a CI will receive a CI regardless whether they participate in this trial or not, since for these patients CI implantation is reimbursed by health insurance. Without this trial, unilateral patients would not have received a CI, since a CI is not reimbursed by health insurance in the Netherlands for unilateral SNHL. Also bilateral patients who already have one CI would not have been reimbursed in most cases for receiving a second CI. Therefore, these patient groups have an added individual patient benefit of having the device with CI functionality (i.e. the cochlear part of the CVI) reimbursed by the trial.

Patients will not receive a benefit from the VI part of the device, since it will only be activated during trial-related activities under supervision in the hospital. Because the trial is designed to first investigate the safety and efficacy of chronic daily vestibular stimulation, no promises can be given on the availability of a CVI/AMP device as part of clinical care. However, it would be possible in principle for a combined processor to be developed as an investigational medical device for take-home use by the patient, as a future extension of this study. Both the manufacturer of the device and the principle investigator intend to pursue this study extension towards home-use when the mid-term results look favorable towards a safe and effective intervention. In other words: the implanted part does not have to be explanted and might still be used in the future.

11.4.2. *Patient group benefits*
BV represents a major handicap with strong balance disturbances, higher risk of falling, visual symptoms (oscillopsia) and a loss of autonomy. There is no therapeutic strategy at the moment. Development of a

vestibular prosthesis could help this group of patients to have a better quality of life. With this study we hope to answer the formerly mentioned crucial questions and to bring essential knowledge towards the development and refining of a vestibular prosthesis.

This trial is part of a larger plan to evaluate the concept of vestibular stimulation to the point that it can be adopted into clinics. As described in chapter 1.4, substantial research has been conducted with VI stimulation. This trial serves as the next step in this process. Alongside this trial a HTA will be performed to also aid in the development of the CVI concept. If this trial succeeds in proving the safety and efficacy of prolonged vestibular stimulation within this patient group, future trials with an extension of the patient cohort are in the pipeline to build on the knowledge gathered in this trial.

11.4.3. *Risk assessment*
See chapter 6.4 and section D of the standard research file for device related risks. See chapter 13 for a general risk assessment.

11.5. Compensation for injury
The sponsor/investigator has a liability insurance which is in accordance with article 7 of the WMO.

The sponsor also has an insurance which is in accordance with the legal requirements in the Netherlands (Article 7 WMO). This insurance provides cover for damage to research subjects through injury or death caused by the study.

1. € 650.000,-- (i.e. six hundred and fifty thousand Euro) for death or injury for each subject who participates in the Research;
2. € 5.000.000,-- (i.e. five million Euro) for death or injury for all subjects who participate in the research combined;
3. € 7.500.000,-- (i.e. seven million five hundred thousand Euro) for the total damage incurred by the organization for all damage disclosed by scientific research for the Sponsor as ‘verrichter’ in the meaning of said Act in each year of insurance coverage.

The insurance applies to the damage that becomes apparent during the study or within 4 years after the end of the study.

11.6. Incentives
Patients with unilateral SNHL and patients with bilateral SNHL and already one CI will receive a CI as part of the CVI which would otherwise not be covered by Dutch health insurance. Patients will be reimbursed for travel costs related to all extra trial visits. Hotel and meal costs during the fitting period and prolonged stimulation period will be paid for. There will not be any other kind of compensation.

12. ADMINISTRATIVE ASPECTS, MONITORING AND PUBLICATION

12.1. Handling and storage of data and documents

Data will be pseudonymized with a code consisting of “CVI” followed by a number. The number is sequentially allocated to each patient at the moment of inclusion (i.e. the first included patient will be CVI-01). The key to the code is safeguarded in a file which is only accessible for the coordinating investigator and the researchers listed on page 2 of this research protocol.

All data will be stored within the academic hospital Maastricht (azM) database. Non-digital data will be stored within the ENT department of the MUMC+. The complete dataset will be accessible only by the researchers of the MUMC+ who participate in the project. Monitors, alongside national and international supervisory authorities, will be allowed to access the data upon request. Also the DSMB described in the DSMB charter (section K5 of the standard research file) will have access to the data, to the extent described in the DSMB charter. Pseudonymized data will be shared with our consortium partners when necessary for analysis. This will include the sharing of data outside of the EU, to our consortium partner in Geneva, Switzerland. The European Committee has decided that Switzerland upholds a suitable protection level for the protection of personal data.

The patient’s name and contact details will be given to the manufacturer (MED-EL) to allow for regular post-implantation surveillance of the implant, conform standard clinical practice. The patient’s name and contact details will also be given to the NH-hotel as described within the patient information (section E1 of the standard research file) to the extent necessary to arrange a hotel booking for the patient. All data transfers will be performed via the guidelines of the MUMC+ regarding safe data transferring. No personal data will be sent outside of the European Union.

Via the informed consent form (see section E2 of the standard research file) the patient will be asked whether their data can be used for future research regarding vestibular implants. Data will not be stored longer than necessary. Data will be handled and stored according to the “General Data Protection Regulation”. Within the Netherlands, data will be handled and stored according to the “Dutch Act on Implementation of the General Data Protection Regulation”. The azM, where this project takes place, maintains strict requirements for ensuring the privacy of patients. A full data management plan will follow.

The study results will be stored for 15 years after study termination, which is in agreement with the “International Conference on Harmonization (ICH)/WMO Good Clinical practice (ICH GCP)”

12.2. Monitoring and Quality Assurance

The monitoring of this study will be performed by the Clinical Trial Center Maastricht (CTCM). The intensity and the content of the monitoring will be determined based on a risk qualification which will be included in a study-specific monitoring plan.

12.3. Amendments

Amendments are changes made to the research after a favorable opinion by the accredited METC has been given. All amendments will be notified to the METC that gave a favorable opinion.

12.4. Annual progress report

The sponsor/investigator will submit a summary of the progress of the trial to the accredited METC once a year. Information will be provided on the date of inclusion of the first subject, numbers of subjects included and numbers of subjects that have completed the trial, serious adverse events/ serious adverse reactions, other problems, and amendments.

12.5. Temporary halt and (prematurely) end of study report

The investigator/sponsor will notify the accredited METC of the end of the study within a period of 8 weeks. The end of the study is defined as the last patient’s last visit.

The sponsor will notify the METC immediately of a temporary halt of the study, including the reason of such an action.

In case the study is ended prematurely, the sponsor will notify the accredited METC within 15 days, including the reasons for the premature termination.

Within one year after the end of the study, the investigator/sponsor will submit a final study report with the results of the study, including any publications/abstracts of the study, to the accredited METC.

12.6. Public disclosure and publication policy

The publication policy conforms to the “CCMO statement on publication policy”. The trial protocol will be registered in clinicaltrials.gov before the first patient is recruited. There are no arrangements made between the sponsor and the investigator concerning the public disclosure and publication of the research data.

13. STRUCTURED RISK ANALYSIS

13.1. Potential issues of concern

13.1.1. *Level of knowledge about mechanism of action*

The mechanism of action is based on an established medical device: the CI. Alternating electrical current generated close to afferent nerve fibers within the inner ear has shown to be effective at eliciting a nerve response [110], and has since been developed into an effective treatment for deafness [111]. The VI is effectively a modified CI, utilizing the same technology and the same type of electrical stimulation. Effective re-establishment of vestibular functions using a VI based on this mechanism of action has been shown by us and by others [13,16,18,20–22,24–26,31]. Both the knowledge about the mechanism of action of the VI and the knowledge and experience of CI technology do not indicate any potential issues of concern for utilizing the CVI in this trial for patients with BV and SNHL in the ear to be implanted (see also chapter 1.4 and 6.4).

13.1.2. *Previous use of the CVI in human beings*

Within the Geneva-Maastricht group, 13 patients have so far been implanted with a CVI device. During these previous experiments, no safety issues were encountered with the CVI. The design of the implant that will be used during this trial is very similar to what was used previously in patients. Also the mechanism of action is the same. The main change between this trial and previous experiments is that a new processor unit (the AMP) will be used to control the device while the patient is under supervision in the hospital. With this new processor, all three electrodes which are implanted into the vestibular system can be stimulated simultaneously, allowing the full range of head motions to be conveyed to the patient via the implant. Also it allows the VI and the CI functionality to be active at the same time. Furthermore, this processor is a wearable, allowing patients to perform daily activities while the CVI is active. All these new features work within the framework of the previously tested VI system and validated CI technology. Therefore these features are not expected to cause any issues of concern regarding patient safety, and the risks for the patient are viewed as similar to receiving a CI.

13.1.3. *Can the mechanisms be induced in animals*

Animal models have been used to great extent by other research groups to establish the mechanism of action for a VI [35,80,112,113]. The principal response to stimulation has been shown to be similar between animals and humans. However, also notable differences have been found in habituation to vestibular stimulation (habituation being faster generally for humans). The animal research has shown the safety of vestibular stimulation, while also indicating the need for testing the implant in humans, due to the complex functional requirements of restoring senses in humans.

13.1.4. *Selectivity of the mechanism*

Due to the CVI being based on electrical stimulation, current spread will occur. As the stimulation current increases, so does current spread, which may lead to stimulation of other nerves such as facial nerves or auditory nerves. The potential for stimulating nerves outside of the inner ear is reduced due to the way the stimulation parameters are chosen, setting the maximum stimulation currents below the current at

which unwanted stimulation occurs (see chapter 8.3.2). This is the same way as current spread is reduced with a CI. Part of the study is to safely investigate the presence of interfering current spread between the cochlea and the vestibular system. The interaction between vestibular and cochlear stimulation might (temporarily) result in a reduced CI or VI performance. The patient will be informed that CI performance might be reduced during simultaneous stimulation, since this might result in a burden to the patient. This will only be the case while the VI-part is active during the VI fitting period and the prolonged stimulation period. While optimizing the VI stimulation response during the VI fitting, the goal is to minimize the interaction between CI and VI stimulation and therefore minimize this burden.

13.1.5. *Analysis of potential effects*

Based on clinical experience with BV and the previous research conducted by the Geneva-Maastricht group, a set of vestibular tests have been chosen to evaluate the vestibular response on several different levels of functionality along the entire natural operating range of the vestibular organ (see chapter 8.1 and 8.3). These tests will allow for the quantification of the efficacy of the prolonged vestibular stimulation. Any potential adverse effects based on current spread will be minimized during the fitting period and its influence on VOR misalignment and hearing will be characterized. Any other adverse effects due to stimulation (i.e. nausea) can potentially be mitigated by optimizing the stimulation parameters and at any time be stopped by turning off the CVI processor or removing the processor from the patient's head.

13.1.6. *Pharmacokinetic considerations*

- Not applicable –

13.1.7. *Study population*

The study population consists of capacitated adults who have disabling symptoms of BV and severe SNHL in at least the ear to be implanted. The condition of these patients is required to be stable and irresponsive to therapy such as physiotherapeutic rehabilitation. Due to the radiation exposure during the trial (imaging), pregnant women are not included into the patient population. The full set of inclusion and exclusion criteria can be found in chapter 4.2 and 4.3.

13.1.8. *Interaction with other products*

At any other point than during the prolonged stimulation period and the VI fitting period, the CVI implant will be driven by a regular CI processor. This CI processor is described in chapter 7. The CI processor will only stimulate the electrodes which are inserted into the cochlea, effectively turning the CVI into a regular CI implant. Stimulation of the CVI implant with a regular CI processor brings with it the same risks that are associated with the use of a regular CI.

13.1.9. *Predictability of the effect*

As shown by the data mentioned in chapter 4.4, the effect of the VI on DVA is expected to be large and a statistically significant difference is expected to be found with the sample size of 8 patients. Also the other outcome measures (e.g. 3D HIT and fHIT) are expected to improve from absent or severely impaired

towards normal responses, as was shown in our previous research within the Geneva-Maastricht group [13,76].

13.1.10. *Can effects be managed*

Any other adverse effects due to stimulation (e.g. nausea) can potentially be mitigated by optimizing the stimulation parameters and at any time be stopped by turning off the CVI processor or removing the processor from the patient's head. Any other adverse effects occurring due to implantation of the device will be dealt with in a similar fashion as is done for a CI (see chapter 6.4.1, 6.4.3 and 6.4.4).

13.1.11. *Risk and justification of perioperative and postoperative radiation use*

During the implantation surgery two radiation based techniques will be utilized to ensure accurate placement of the electrodes into the SCCs: neuronavigation and fluoroscopy. Previous research has shown the importance of accurate electrode placement on the functioning of a VI, indicating that electrode misplacement by just a few millimeters causes a substantial increase in the minimum current necessary for stimulating the vestibular nerves [94]. With accurate placement, the stimulation currents necessary to generate nerve responses can be substantially lower, reducing the problems caused by current spread both on VOR misalignment and CI performance. Lower minimal stimulation currents also allow for better modulation of the stimulation signal and prolonged battery life of the AMP. A 'blind' insertion, i.e. without supportive imaging techniques, results in electrodes that are on average placed a considerable distance away from the SCC ampulla, resulting in only 75% of the electrodes being placed within a tolerable distance to the target location [93]. To establish a correct electrode placement, both the fenestrations in the SCCs and the electrode insertion depth into the SCCs need to be accurate. Within our group, a surgical technique has been developed to allow for this.

In standard clinical practice, patients that undergo retro-auricular ear surgery receive a preoperative CT scan of the mastoid to inform the surgeon about individual anatomy. We will perform the same pre-operative scan used in standard clinical CI practice as part of the pre-operative assessment for our VCI surgeries. This pre-operative CT will also be fused with the 3D-CT to assess electrode placement per-operative (cfr. Infra). The resulting radiation dose from this CT scan is 0.6 mSv.To assess the right insertion depth of the electrodes into the SCCs, a fluoroscopy-based technique is used. Fluoroscopy consists of making real-time x-ray images of, in this case, the inner ear. Within the image created by using fluoroscopy, the electrodes are visualized within the SCCs in real-time, allowing for fine-tuning the placement of the electrodes in the SCCs. In previous research, the fluoroscopy-based technique was developed and optimized, resulting in an efficient work-flow only requiring between 0.019 and 0.042 mSv to achieve a significant improvement in electrode placement accuracy [93]. Within this pilot study, a substantial improvement was achieved over "blind" insertion, resulting in 94% of the electrodes being placed within the desired distance to the target.

After using the 2D guidance of fluoroscopy to insert the electrodes in the SCC's, an intra-operative 3D-CT of the head will be made to verify the exact location of the electrodes (distance to the ampulla) in 3 dimensions. We namely will fuse the 3D-CT image onsite with the pre-operative CT mastoid (free of scattering by the electrodes), allowing us to improve the accuracy of electrode placement even further. If

deemed necessary, repositioning of the electrodes will be performed using fluoroscopy guidance and if necessary second 3D-CT is used to register the position of the repositioned electrodes. A final 3DCT is made after fixation of the electrodes. The radiation dose of the intra-operative 3D-CT has been calculated and was found to be low, only 0.19 mSv per scan.

Postoperatively and at 1, 2, and 5 years follow-up, a low-dose CBCT scan will be made to check the position of the electrodes in the cochlea and SCCs. In other clinics, this is often already a standard procedure after cochlear implantation. In our clinic, a post-operative CBCT scan is often included when clinical research is performed involving CI's ("ELEPHANT Trial" ABR 51559, "Bimodal Zoom" ABR 64874). Also within clinical care it is generally applied when problems or complaints occur with regular CI devices. The CBCT scan involves only a very small dose of radiation (0.05 mSv). It will allow for accurate assessment of the placement of electrodes in both the SCCs and the cochlea. This information can then be linked to device efficacy and it can be used to assess potential causes of problems with the CVI if they occur. It will also allow for evaluating the benefit from postoperative scanning in the follow-up of a CVI.

Table 7 Overview of radiation doses during the trial

	**Minimum (mSv)**	**Maximum (mSv)**
Pre-operative CT	0.60	0.60
Peri-operative fluoroscopy	0.02	0.08 (possible extra fluoroscopy after 3DCT)
Peri-operative 3D-CT	0.38 (2 x 0.19)	0.57 (3 x 0.19)
Post-operative CBCT	0.20 (4 x 0.05)	0.20 (4 x 0.05)
Total radiation exposure	1.20	1.45
Extra exposure as part of the trial (Total – Pre-op CT)	0.60	0.85

The total radiation exposure for patients included in this trial is between 1.20 and 1.41 mSv (table 7). Since performing a pre-operative CT is part of the standard clinical CI work-up, this means that there is an increased radiation exposure of 0.60-0.81 mSv (depending on the number of 3D-CT's needed intra-operatively) for receiving a CVI compared to receiving a regular CI (regular exposure is around 0.6 mSv). This places the extra radiation exposure associated with this trial in risk category IIa of the International Committee of Radiological Protection (ICRP) (between 0.1 and 1 mSv). For all patients there is a therapeutic effect by taking part in the trial: the CI part of the CVI can be used daily and will lead to increased hearing performance and thus a direct therapeutic effect.

13.1.12. *Risks related to the tests included in the trial*

The tests included in this study are: VOR tests, VSR tests, tests of vestibular perception, a gait analysis, hearing tests and questionnaires. All these tests are routinely used in our clinic and research practices, and have proven to be safe [21,76,114].

Semi-structured qualitative interviews will be conducted and involve patient expectation and patient experiences when being part of the trial. The team of researchers will explicitly consider the personal limits of the patients, as will be done during all trial related activities.

The tests included in this study are: VOR tests, VSR tests, tests of vestibular perception, a gait analysis, hearing tests and questionnaires. All these tests are routinely used in our clinic and research practices, and have proven to be safe [21,76,114].

13.1.13. *Risks related to the COVID-19 epidemic*

Due to the uncertainty of how long the government-mandated security measures to prevent the spread of the COVID-19 virus will maintain active, all trial related procedures will be made COVID-19 safe for as long as necessary. The COVID-19 rules set up by the MUMC+ will be used as a guideline in order to maintain a safe environment for both patients and staff. For most of the trial activities (especially during the first year of the trial) including tests, surgery and CI rehabilitation there are already COVID-19 safe procedures in place. For trial-specific procedures such as during the fitting and prolonged stimulation periods, standard operating protocols will be written which ensure a safe distance of 1,5 meters between the different people involved and/or provide personal protective equipment (facemasks, disinfectants) to prevent spreading of the virus. Conforming to MUMC+ policy, it will be ensured that the execution of this trial will not have an influence on the regular clinical care which is provided by the hospital.

Decisions on starting, stopping and restarting of the trial will be based on the MUMC+ guidelines. If it were to happen that medical research is not allowed for a certain period due to a wave of infections, or a patient is required to be in home-quarantine at the time a trial activity is planned, this will never lead to an unsafe situation for the patient. None of the trial-related activities which are not also part of clinical care are essential for maintaining the patient’s safety. Therefore all these activities can be postponed without safety-concerns.

The only risk related to a change in schedule due to COVID-19 would be that data-quality might be negatively affected. Since most time points in the study are flexible, this will only be a problem if it occurs during a prolonged stimulation period, which will be at least 1,5 years after the start of the trial. By that time it is highly likely that either a vaccine or a treatment will have been developed which removes the risk of such a temporary halt influencing data quality.

13.2. Synthesis

The main risks which are associated with the implantation and use of the CVI are associated with damaging the sensory structures in the inner ear, resulting in reduced vestibular and auditory response. These risks are mitigated by implanting only patients with severe BV and severe SNHL in the ear to be implanted, meaning these patients have almost no residual function left which can be damaged.

Any other risks associated with the CVI, as described in chapter 6.4 and 13.1, are minimal and similar to receiving a CI. Any risks associated with using the VI part of the CVI are reduced by allowing the stimulation to be stopped immediately by the patient themselves by turning off the AMP or removing the transmitting coil from their head. Alongside this, there will always be a member of the research team present when the VI part of the CVI is active.

According to our opinion, the risk mitigation strategies implemented in the trial result in acceptable risks for the patients participating in this trial.

14. REFERENCES

1. Guyot, J. P. [Review of anatomy and physiology of the vestibular system]. *Rev. Med. Suisse Romande* **113**, 665–669 (1993).
2. Daroff, R. B. Disorders of the Vestibular System. *Neurology* **47**, 1615 LP – 1615 (1996).
3. Ewald, J. R. Zur Physiologie der Bogengänge. *Arch. für die gesamte Physiol. des Menschen und der Tiere* **41**, 463–483 (1887).
4. Guyot, J.-P. & Crescentino, V. [Today's tests of vestibular function]. *Rev. Med. Suisse* **4**, 2085–2088 (2008).
5. Kingma, H. Function tests of the otolith or statolith system. *Curr. Opin. Neurol.* **19**, 21–25 (2006).
6. Rauch, S. D. Vestibular evoked myogenic potentials. *Curr. Opin. Otolaryngol. Head Neck Surg.* **14**, 299–304 (2006).
7. Curthoys, I. S. & Halmagyi, G. M. Vestibular compensation. *Adv. Otorhinolaryngol.* **55**, 82–110 (1999).
8. Ward BK, Agrawal Y, Hoffman HJ, Carey JP, D. S. C. Prevalence and Impact of Bilateral Vestibular Deficiency (BVD): Results from the 2008 United States National Health Interview Survey. *JAMA Otolaryngol Head Neck Surg* **139**, 803–810 (2013).
9. Van De Berg, R., Van Tilburg, M. & Kingma, H. Bilateral Vestibular Hypofunction: Challenges in Establishing the Diagnosis in Adults. *Orl* **77**, 197–218 (2015).
10. Sun, D. Q., Ward, B. K., Semenov, Y. R., Carey, J. P. & Della Santina, C. C. Bilateral vestibular deficiency: Quality of life and economic implications. *JAMA Otolaryngol. - Head Neck Surg.* **140**, 527–534 (2014).
11. Kingma, H. *et al.* Vibrotactile feedback improves balance and mobility in patients with severe bilateral vestibular loss. *J. Neurol.* **266**, 19–26 (2019).
12. Schniepp, R. *et al.* Noisy vestibular stimulation improves vestibulospinal function in patients with bilateral vestibulopathy. *J. Neurol.* **265**, 57–62 (2018).
13. Guinand, N. *et al.* Vestibular Implants: 8 Years of Experience with Electrical Stimulation of the Vestibular Nerve in 11 Patients with Bilateral Vestibular Loss. *Orl* **77**, 227–240 (2015).
14. Kos, M. I. *et al.* Transcanal Approach to the Singular Nerve. *Otol. Neurotol.* **27**, (2006).
15. Feigl, G. C. *et al.* Superior Vestibular Neurectomy: A Novel Transmeatal Approach for a Denervation of the Superior and Lateral Semicircular Canals. *Otol. Neurotol.* **30**, (2009).
16. van de Berg, R., Guinand, N., Guyot, J. P., Kingma, H. & Stokroos, R. J. The modified ampullar approach for vestibular implant surgery: Feasibility and its first application in a human with a long-term vestibular loss. *Front. Neurol.* **FEB**, 1–7 (2012).
17. Guyot, J.-P., Sigrist, A., Pelizzone, M. & Kos, M. I. Adaptation to Steady-State Electrical Stimulation of the Vestibular System in Humans. *Ann. Otol. Rhinol. Laryngol.* **120**, 143–149 (2011).
18. Nguyen, T. A. K. *et al.* Characterization of cochlear, vestibular and cochlear-vestibular electrically evoked compound action potentials in patients with a vestibulo-cochlear implant. *Front. Neurosci.* **11**, 1–12 (2017).
19. van de Berg, R. *et al.* The vestibular implant: Hearing preservation during intralabyrinthine electrode insertion-A case report. *Front. Neurol.* **8**, 1–7 (2017).
20. Fornos, A. P. *et al.* Artificial balance: Restoration of the vestibulo-ocular reflex in humans with a prototype vestibular neuroprosthesis. *Front. Neurol.* **5 APR**, 1–11 (2014).
21. Guinand, N. *et al.* The video head impulse test to assess the efficacy of vestibular implants in humans. *Front. Neurol.* **8**, (2017).
22. van de Berg, R. *et al.* The vestibular implant: Frequency-dependency of the electrically evoked vestibulo-ocular reflex in humans. *Front. Syst. Neurosci.* **8**, 1–12 (2015).
23. Nguyen, T. A. K. *et al.* Characterization of pulse amplitude and pulse rate modulation for a human

vestibular implant during acute electrical stimulation. *J. Neural Eng.* **13**, (2016).
24. van de Berg, R. *et al.* The vestibular implant input interacts with residual natural function. *Front. Neurol.* **8**, (2017).
25. Guinand, N. *et al.* Restoring visual acuity in dynamic conditions with a vestibular implant. *Front. Neurosci.* **10**, 1–6 (2016).
26. DiGiovanna, J., Nguyen, T. A. K., Guinand, N., Pérez-Fornos, A. & Micera, S. Neural Network Model of Vestibular Nuclei Reaction to Onset of Vestibular Prosthetic Stimulation. *Front. Bioeng. Biotechnol.* **4**, 34 (2016).
27. Lucieer, F. *et al.* Bilateral vestibular hypofunction: Insights in etiologies, clinical subtypes, and diagnostics. *Front. Neurol.* **7**, 1–11 (2016).
28. Strupp, M. *et al.* Bilateral vestibulopathy: Diagnostic criteria consensus document of the classification committee of the barany society. *J. Vestib. Res. Equilib. Orientat.* **27**, 177–189 (2017).
29. Lucieer, F. *et al.* Full spectrum of reported symptoms of bilateral vestibulopathy needs further investigation-A systematic review. *Front. Neurol.* **9**, 1–7 (2018).
30. Lucieer, F. M. P. *et al.* Bilateral vestibulopathy: beyond imbalance and oscillopsia. *J. Neurol.* (2020) doi:10.1007/s00415-020-10243-5.
31. Rubinstein, J. T., Ling, L., Nowack, A., Nie, K. & Phillips, J. O. Results From a Second-Generation Vestibular Implant in Human Subjects : Diagnosis May Impact Electrical Sensitivity of Vestibular Afferents. (2019) doi:10.1097/MAO.0000000000002463.
32. Boutros, P. J. *et al.* Continuous vestibular implant stimulation partially restores eye-stabilizing reflexes. *JCI Insight* **4**, (2019).
33. Ramos Macias, A., Ramos De Miguel, A., Rodriguez Montesdeoca, I., Borkoski Barreiro, S. & Falcón González, J. C. Chronic Electrical Stimulation of the Otolith Organ: Preliminary Results in Humans with Bilateral Vestibulopathy and Sensorineural Hearing Loss. *Audiol. Neurotol.* (2019) doi:10.1159/000503600.
34. Phillips, J. O. *et al.* Vestibular implantation and longitudinal electrical stimulation of the semicircular canal afferents in human subjects. *J. Neurophysiol.* **113**, 3866–92 (2015).
35. Dai, C. *et al.* Directional plasticity rapidly improves 3D vestibulo-ocular reflex alignment in monkeys using a multichannel vestibular prosthesis. *JARO - J. Assoc. Res. Otolaryngol.* **14**, 863–877 (2013).
36. Gajadeera, E. A., Galvin, K. L., Dowell, R. C. & Busby, P. A. Gajadeera et al 2017 The change in electrical stimulation levels during 24 months postimplantation for a large cohort of adults.pdf. 357–367 (2017).
37. Sluydts, M. *et al.* Electrical Vestibular Stimulation in Humans: A Narrative Review. *Audiol. Neurotol.* (2019) doi:10.1159/000502407.
38. Sulway, S. & Whitney, S. L. Advances in Vestibular Rehabilitation. in *Advances in Oto-Rhino-Laryngology* vol. 82 164–169 (2019).
39. Whitney, S. L., Sparto, P. J. & Furman, J. M. Vestibular Rehabilitation and Factors That Can Affect Outcome. *Semin. Neurol.* **online 30.**, (2019).
40. Zeh, R. & Baumann, U. [Inpatient rehabilitation of adult CI users: Results in dependency of duration of deafness, CI experience and age]. *HNO* **63**, 557–576 (2015).
41. Phillips, J. O. & Nowack, A. Interactions between Auditory and Vestibular Modalities during Stimulation with a Combined Vestibular and Cochlear Prosthesis. (2020) doi:10.1159/000503846.
42. Holladay, J. T. Proper method for calculating average visual acuity. *J. Refract. Surg.* **13**, 388–391 (1997).
43. Lamberink, H. J. *et al.* Statistical power of clinical trials increased while effect size remained stable: an empirical analysis of 136,212 clinical trials between 1975 and 2014. *J. Clin. Epidemiol.* **102**, 123–128 (2018).

44. Cohen, J. CHAPTER 1 - The Concepts of Power Analysis. in (ed. Cohen, J. B. T.-S. P. A. for the B. S.) 1–17 (Academic Press, 1977). doi:https://doi.org/10.1016/B978-0-12-179060-8.50006-2.
45. Faul, F., Erdfelder, E., Lang, A.-G. & Buchner, A. G*Power 3: a flexible statistical power analysis program for the social, behavioral, and biomedical sciences. *Behav. Res. Methods* **39**, 175–191 (2007).
46. Dhanasingh, A. & Jolly, C. An overview of cochlear implant electrode array designs. *Hear. Res.* **356**, 93–103 (2017).
47. Lazard, D. S. *et al.* Pre-, Per- and Postoperative Factors Affecting Performance of Postlinguistically Deaf Adults Using Cochlear Implants: A New Conceptual Model over Time. *PLoS One* **7**, e48739 (2012).
48. Fishman, K. E., Shannon, R. V & Slattery, W. H. Speech recognition as a function of the number of electrodes used in the SPEAK cochlear implant speech processor. *J. Speech. Lang. Hear. Res.* **40**, 1201–1215 (1997).
49. Shannon, R. V, Zeng, F.-G., Kamath, V., Wygonski, J. & Ekelid, M. Speech Recognition with Primarily Temporal Cues. *Science (80-.).* **270**, 303–304 (1995).
50. Perreau, A., Tyler, R. S. & Witt, S. A. The effect of reducing the number of electrodes on spatial hearing tasks for bilateral cochlear implant recipients. *J. Am. Acad. Audiol.* **21**, 110–120 (2010).
51. Friesen, L. M., Shannon, R. V, Baskent, D. & Wang, X. Speech recognition in noise as a function of the number of spectral channels: comparison of acoustic hearing and cochlear implants. *J. Acoust. Soc. Am.* **110**, 1150–1163 (2001).
52. Riss, D., Arnoldner, C., Baumgartner, W.-D., Kaider, A. & Hamzavi, J.-S. A new fine structure speech coding strategy: speech perception at a reduced number of channels. *Otol. Neurotol. Off. Publ. Am. Otol. Soc. Am. Neurotol. Soc. [and] Eur. Acad. Otol. Neurotol.* **29**, 784–788 (2008).
53. Canfarotta, M. W. *et al.* Frequency-to-Place Mismatch: Characterizing Variability and the Influence on Speech Perception Outcomes in Cochlear Implant Recipients. *Ear Hear.* **41**, 1349–1361 (2020).
54. O'Connell, B. P. *et al.* Electrode Location and Angular Insertion Depth Are Predictors of Audiologic Outcomes in Cochlear Implantation. *Otol. Neurotol. Off. Publ. Am. Otol. Soc. Am. Neurotol. Soc. [and] Eur. Acad. Otol. Neurotol.* **37**, 1016–1023 (2016).
55. Canfarotta, M. W. *et al.* Long-Term Influence of Electrode Array Length on Speech Recognition in Cochlear Implant Users. *Laryngoscope* **n/a**,.
56. van der Marel, K. S., Briaire, J. J., Verbist, B. M., Muurling, T. J. & Frijns, J. H. M. The influence of cochlear implant electrode position on performance. *Audiol. Neurootol.* **20**, 202–211 (2015).
57. Radeloff, A., Mack, M., Baghi, M., Gstoettner, W. K. & Adunka, O. F. Variance of angular insertion depths in free-fitting and perimodiolar cochlear implant electrodes. *Otol. Neurotol. Off. Publ. Am. Otol. Soc. Am. Neurotol. Soc. [and] Eur. Acad. Otol. Neurotol.* **29**, 131–136 (2008).
58. Adunka, O. F., Pillsbury, H. C., Adunka, M. C. & Buchman, C. A. Is electric acoustic stimulation better than conventional cochlear implantation for speech perception in quiet? *Otol. Neurotol. Off. Publ. Am. Otol. Soc. Am. Neurotol. Soc. [and] Eur. Acad. Otol. Neurotol.* **31**, 1049–1054 (2010).
59. Brant, J. A. & Ruckenstein, M. J. Electrode selection for hearing preservation in cochlear implantation: A review of the evidence. *World J. Otorhinolaryngol. - head neck Surg.* **2**, 157–160 (2016).
60. SUZUKI, J. I., COHEN, B. & BENDER, M. B. COMPENSATORY EYE MOVEMENTS INDUCED BY VERTICAL SEMICIRCULAR CANAL STIMULATION. *Exp. Neurol.* **9**, 137–160 (1964).
61. COHEN, B. & SUZUKI, J. I. Eye movements induced by ampullary nerve stimulation. *Am. J. Physiol.* **204**, 347–351 (1963).
62. Davidovics, N. S., Fridman, G. Y., Chiang, B. & Della Santina, C. C. Effects of Biphasic Current Pulse Frequency, Amplitude, Duration, and Interphase Gap on Eye Movement Responses to Prosthetic

Electrical Stimulation of the Vestibular Nerve. *IEEE Trans. Neural Syst. Rehabil. Eng.* **19**, 84–94 (2011).
63. Lewis, R. F., Haburcakova, C., Gong, W., Makary, C. & Merfeld, D. M. Vestibuloocular reflex adaptation investigated with chronic motion-modulated electrical stimulation of semicircular canal afferents. *J. Neurophysiol.* **103**, 1066–1079 (2010).
64. Lewis, R. F., Haburcakova, C., Gong, W., Karmali, F. & Merfeld, D. M. Spatial and temporal properties of eye movements produced by electrical stimulation of semicircular canal afferents. *J. Neurophysiol.* **108**, 1511–1520 (2012).
65. Fridman, G. Y., Davidovics, N. S., Dai, C., Migliaccio, A. A. & Della Santina, C. C. Vestibulo-ocular reflex responses to a multichannel vestibular prosthesis incorporating a 3D coordinate transformation for correction of misalignment. *JARO - J. Assoc. Res. Otolaryngol.* **11**, 367–381 (2010).
66. Dai, C. *et al.* Cross-axis adaptation improves 3D vestibulo-ocular reflex alignment during chronic stimulation via a head-mounted multichannel vestibular prosthesis. *Exp. Brain Res.* **210**, 595–606 (2011).
67. Dai, C. *et al.* Restoration of 3D vestibular sensation in rhesus monkeys using a multichannel vestibular prosthesis. *Hear. Res.* **281**, 74–83 (2011).
68. Davidovics, N. S., Fridman, G. Y. & Della Santina, C. C. Co-modulation of stimulus rate and current from elevated baselines expands head motion encoding range of the vestibular prosthesis. *Exp. brain Res.* **218**, 389–400 (2012).
69. Lewis, R. F. *et al.* Vestibular adaptation studied with a prosthetic semicircular canal. *J. Vestib. Res.* **12**, 87–94.
70. Merfeld, D. M. *et al.* Acclimation to chronic constant-rate peripheral stimulation provided by a vestibular prosthesis. *IEEE Trans. Biomed. Eng.* **53**, 2362–2372 (2006).
71. Merfeld, D. M., Haburcakova, C., Gong, W. & Lewis, R. F. Chronic vestibulo-ocular reflexes evoked by a vestibular prosthesis. *IEEE Trans. Biomed. Eng.* **54**, 1005–1015 (2007).
72. Gong, W., Haburcakova, C. & Merfeld, D. M. Vestibulo-ocular responses evoked via bilateral electrical stimulation of the lateral semicircular canals. *IEEE Trans. Biomed. Eng.* **55**, 2608–2619 (2008).
73. Thompson, L. A. *et al.* Responses evoked by a vestibular implant providing chronic stimulation. *J. Vestib. Res. Equilib. Orientat.* **22**, 11–15 (2012).
74. Lewis, R. F., Haburcakova, C., Gong, W., Lee, D. & Merfeld, D. Electrical stimulation of semicircular canal afferents affects the perception of head orientation. *J. Neurosci.* **33**, 9530–9535 (2013).
75. Mitchell, D. E. *et al.* Head Movements Evoked in Alert Rhesus Monkey by Vestibular Prosthesis Stimulation: Implications for Postural and Gaze Stabilization. *PLoS One* **8**, 1–12 (2013).
76. Starkov, D. *et al.* Restoring the High-Frequency Dynamic Visual Acuity with a Vestibular Implant Prototype in Humans. *Audiol. Neurotol.* (2019) doi:10.1159/000503677.
77. Cohen, N. L. & Hoffman, R. A. Complications of Cochlear Implant Surgery in Adults and Children. *Ann. Otol. Rhinol. Laryngol.* **100**, 708–711 (1991).
78. Arts, R. A. G. J. *et al.* Tinnitus Suppression by Intracochlear Electrical Stimulation in Single Sided Deafness - A Prospective Clinical Trial: Follow-Up. *PLoS One* **11**, e0153131 (2016).
79. Battmer, R.-D., Linz, B. & Lenarz, T. A Review of Device Failure in More Than 23 Years of Clinical Experience of a Cochlear Implant Program With More Than 3,400 Implantees. *Otol. Neurotol.* **30**, (2009).
80. Gong, W. & Merfeld, D. M. Prototype Neural Semicircular Canal Prosthesis using Patterned Electrical Stimulation. *Ann. Biomed. Eng.* **28**, 572–581 (2000).
81. Wall, C., Kos, M. I. & Guyot, J. P. Eye movements in response to electric stimulation of the human

posterior ampullary nerve. *Ann. Otol. Rhinol. Laryngol.* **116**, 369–374 (2007).
82. Lim, C.-Y. & In, J. Randomization in clinical studies. *Korean J. Anesthesiol.* **72**, 221–232 (2019).
83. Shepard, N. T. & Jacobson, G. P. The caloric irrigation test. *Handb. Clin. Neurol.* **137**, 119–131 (2016).
84. Senden, R., Savelberg, H. H. C. M., Grimm, B., Heyligers, I. C. & Meijer, K. Accelerometry-based gait analysis, an additional objective approach to screen subjects at risk for falling. *Gait Posture* **36**, 296–300 (2012).
85. Sibley, K. M. *et al.* Recommendations for a core outcome set for measuring standing balance in adult populations: a consensus-based approach. *PLoS One* **10**, e0120568–e0120568 (2015).
86. Franchignoni, F., Horak, F., Godi, M., Nardone, A. & Giordano, A. Using psychometric techniques to improve the Balance Evaluation Systems Test: the mini-BESTest. *J. Rehabil. Med.* **42**, 323–331 (2010).
87. Bosman, A. J. & Smoorenburg, G. F. Intelligibility of Dutch CVC syllables and sentences for listeners with normal hearing and with three types of hearing impairment. *Audiology* **34**, 260–284 (1995).
88. van Wieringen, A. & Wouters, J. LIST and LINT: sentences and numbers for quantifying speech understanding in severely impaired listeners for Flanders and the Netherlands. *Int. J. Audiol.* **47**, 348–355 (2008).
89. Theelen - van den Hoek, F., Houben, R. & Dreschler, W. Investigation into the applicability and optimization of the Dutch matrix sentence test for use with cochlear implant users. *Int. J. Audiol.* **53**, 1–12 (2014).
90. Wiltfang, J. *et al.* Intraoperative Image-Guided Surgery of the Lateral and Anterior Skull Base in Patients with Tumors or Trauma. *Skull Base* **13**, 21–29 (2003).
91. Kral, F., Riechelmann, H. & Freysinger, W. Navigated surgery at the lateral skull base and registration and preoperative imagery: experimental results. *Arch. Otolaryngol. Head. Neck Surg.* **137**, 144–150 (2011).
92. Komune, N. *et al.* The accuracy of an electromagnetic navigation system in lateral skull base approaches. *Laryngoscope* **127**, 450–459 (2017).
93. Stultiens, J. J. A. *et al.* Vestibular Implantation and the Feasibility of Fluoroscopy-Guided Electrode Insertion. *Otolaryngol. Clin. North Am.* **53**, 115–126 (2020).
94. Hedjoudje, A. *et al.* Virtual Rhesus Labyrinth Model Predicts Responses to Electrical Stimulation Delivered by a Vestibular Prosthesis. *J. Assoc. Res. Otolaryngol.* **20**, 313–339 (2019).
95. Kempen, G., Zijlstra, G. A. & van Haastregt, J. Het meten van angst om te vallen met de Falls Efficacy Scale-International (FES-I). Achtergrond en psychometrische kenmerken. *Tijdschr. voor Gerontol. en Geriatr. 2007* **38**, 178–184 (2007).
96. Vereeck, L., Truijen, S., Wuyts, F. & Van de Heyning, P. H. Test-retest reliability of the Dutch version of the Dizziness Handicap Inventory. *B-ENT* **2**, 75–80 (2006).
97. Guinand, N., Pijnenburg, M., Janssen, M. & Kingma, H. Visual acuity while walking and oscillopsia severity in healthy subjects and patients with unilateral and bilateral vestibular function loss. *Arch. Otolaryngol. Head. Neck Surg.* **138**, 301–306 (2012).
98. Anson, E. R., Gimmon, Y., Kiemel, T., Jeka, J. J. & Carey, J. P. A Tool to Quantify the Functional Impact of Oscillopsia. *Front. Neurol.* **9**, 142 (2018).
99. Furlong, W. J., Feeny, D. H., Torrance, G. W. & Barr, R. D. The Health Utilities Index (HUI) system for assessing health-related quality of life in clinical studies. *Ann. Med.* **33**, 375–384 (2001).
100. M Versteegh, M. *et al.* Dutch Tariff for the Five-Level Version of EQ-5D. *Value Heal. J. Int. Soc. Pharmacoeconomics Outcomes Res.* **19**, 343–352 (2016).
101. Al-Janabi, H., Flynn, T. N. & Coast, J. Development of a self-report measure of capability wellbeing for adults: the ICECAP-A. *Qual. life Res. an Int. J. Qual. life Asp. Treat. care Rehabil.* **21**, 167–176

(2012).
102. SPINHOVEN, P. H. *et al.* A validation study of the Hospital Anxiety and Depression Scale (HADS) in different groups of Dutch subjects. *Psychol. Med.* **27**, 363–370 (1997).
103. Noble, W., Jensen, N. S., Naylor, G., Bhullar, N. & Akeroyd, M. A. A short form of the Speech, Spatial and Qualities of Hearing scale suitable for clinical use: the SSQ12. *Int. J. Audiol.* **52**, 409–412 (2013).
104. Gatehouse, S. & Noble, W. The Speech, Spatial and Qualities of Hearing Scale (SSQ). *Int. J. Audiol.* **43**, 85–99 (2004).
105. Meeus, O., Blaivie, C. & Van de Heyning, P. Validation of the Dutch and the French version of the Tinnitus Questionnaire. *B-ENT* **3 Suppl 7**, 11–17 (2007).
106. Kloostra, F. J. J. *et al.* A Prospective Study of the Effect of Cochlear Implantation on Tinnitus. *Audiol. Neurotol.* **23**, 356–363 (2018).
107. Arts, R. A. G. J., Netz, T., Janssen, A. M. L., George, E. L. J. & Stokroos, R. J. The occurrence of tinnitus after CI surgery in patients with severe hearing loss: A retrospective study. *Int. J. Audiol.* **54**, 910–917 (2015).
108. Nicholas, P., Hefford, C. & Tumilty, S. The use of the Patient-Specific Functional Scale to measure rehabilitative progress in a physiotherapy setting. *J. Man. Manip. Ther.* **20**, 147–152 (2012).
109. Calmettes, G., Drummond, G. B. & Vowler, S. L. Making do with what we have: use your bootstraps. *Br. J. Pharmacol.* **167**, 233–237 (2012).
110. DJOURNO, A. & EYRIES, C. [Auditory prosthesis by means of a distant electrical stimulation of the sensory nerve with the use of an indwelt coiling]. *Presse Med.* **65**, 1417 (1957).
111. Eshraghi, A. A. *et al.* The cochlear implant: historical aspects and future prospects. *Anat. Rec. (Hoboken).* **295**, 1967–1980 (2012).
112. Hageman, K. N. *et al.* Binocular 3D otolith-ocular reflexes: responses of chinchillas to prosthetic electrical stimulation targeting the utricle and saccule. *J. Neurophysiol.* **123**, 259–276 (2019).
113. Sun, D. Q. *et al.* Chronic stimulation of the semicircular canals using a multichannel vestibular prosthesis: Effects on locomotion and angular vestibulo-ocular reflex in chinchillas. *Proc. Annu. Int. Conf. IEEE Eng. Med. Biol. Soc. EMBS* 3519–3523 (2011) doi:10.1109/IEMBS.2011.6090584.
114. McCrum, C. *et al.* The walking speed-dependency of gait variability in bilateral vestibulopathy and its association with clinical tests of vestibular function. *Sci. Rep.* **9**, 18392 (2019).115. Luts H, Jansen S, Dreschler W, Wouters J, editors. Development and normative data for the Flemish/Dutch Matrix test (2014).
116. Smits C, Theo Goverts S, Festen JM. The digits-in-noise test: assessing auditory speech recognition abilities in noise. J Acoust Soc Am. 2013;133(3):1693-706.
117. Hall J. New handbook of auditory evoked responses. Boston: Pearson; 2007.
